# Up-Regulation of Tumor Necrosis Factor Superfamily Genes in Early Phases of Photoreceptor Degeneration

**DOI:** 10.1371/journal.pone.0085408

**Published:** 2013-12-19

**Authors:** Sem Genini, William A. Beltran, Gustavo D. Aguirre

**Affiliations:** Section of Ophthalmology, Department of Clinical Studies, School of Veterinary Medicine, University of Pennsylvania, Philadelphia, Pennsylvania, United States of America; Justus-Liebig-University Giessen, Germany

## Abstract

We used quantitative real-time PCR to examine the expression of 112 genes related to retinal function and/or belonging to known pro-apoptotic, cell survival, and autophagy pathways during photoreceptor degeneration in three early-onset canine models of human photoreceptor degeneration, rod cone dysplasia 1 (rcd1), X-linked progressive retinal atrophy 2 (xlpra2), and early retinal degeneration (erd), caused respectively, by mutations in *PDE6B*, *RPGR*ORF15, and *STK38L*. Notably, we found that expression and timing of differentially expressed (DE) genes correlated with the cell death kinetics. Gene expression profiles of rcd1 and xlpra2 were similar; however rcd1 was more severe as demonstrated by the results of the TUNEL and ONL thickness analyses, a greater number of genes that were DE, and the identification of altered expression that occurred at earlier time points. Both diseases differed from erd, where a smaller number of genes were DE. Our studies did not highlight the potential involvement of mitochondrial or autophagy pathways, but all three diseases were accompanied by the down-regulation of photoreceptor genes, and up-regulation of several genes that belong to the TNF superfamily, the extrinsic apoptotic pathway, and pro-survival pathways. These proteins were expressed by different retinal cells, including horizontal, amacrine, ON bipolar, and Müller cells, and suggest an interplay between the dying photoreceptors and inner retinal cells. Western blot and immunohistochemistry results supported the transcriptional regulation for selected proteins. This study highlights a potential role for signaling through the extrinsic apoptotic pathway in early cell death events and suggests that retinal cells other than photoreceptors might play a primary or bystander role in the degenerative process.

## Introduction

The visual process is initiated by quantal light absorption by opsin visual pigments and signal generation, first in the photoreceptor (PR) cells and subsequently through two complex synaptic pathways in the outer and inner plexiform layers to convey the information to higher visual centers. The intricate structure of highly differentiated PRs is ideally suited for light absorption and signal transduction, and is highly dependent on the expression of multiple genes involved, first in PR specification, and then differentiation and maintenance [1]. In humans, 232 loci are associated with retinal degeneration; of these 192 have been identified as the causative genes (RetNet: http://www.sph.uth.tmc.edu/RetNet/; June 2013). A comparable, albeit smaller, number of genes are associated with retinal degeneration in animals [2,3]. 

PR death is a common fate in retinal degenerations (reviewed by [4]) and occurs through a variety of molecular mechanisms and in response to multiple genetic or acquired insults [5]. Disease-associated PR cell death has been reported to occur via apoptosis [6,7], and several studies have implicated various mechanisms and pathways involved in retinal cell death [8-12]. A compensatory but imperfect survival response also occurs [12,13]. Disease-specific cell death and survival responses depend on the underlying mutation, whether the disease occurs naturally or is induced, the speed of the degenerative process, the cell types involved, and many other factors [10,12]. 

Although the causative genetic mutations resulting in PR disease are often well characterized, the molecular events that link the mutation to cell death are still unknown. Indeed, because of experimental limitations when working with human subjects, e.g. lack of adequate sample numbers at the appropriate disease stages, studies of retinal degeneration mechanisms rely heavily on the use of corresponding animal models. In dogs, inherited retinopathies occur and result from mutations in multiple genes [3]. In the present study we used three canine retinal disease models-rcd1, xlpra2, and erd-to examine the molecular mechanisms of cell death. Both rcd1 [14,15] and xlpra2 [16] bear mutations in genes, rod cyclic GMP phosphodiesterase ß subunit (*PDE6B*) and retinitis pigmentosa GTPase regulator (*RPGR*), respectively, that cause human inherited blindness, and the disease phenotypes are similar and comparable. For erd (Serine/Threonine Kinase 38 Like*/ STK38L*-mutant), no equivalent disease has been reported in humans [17]. In all three, the early and rapid degeneration of the PRs makes the disease course predictable and highly suitable for comparative studies of the degenerative events. The exact mechanisms by which mutations in these genes drive the disease progression are currently unknown. As a first step in trying to characterize disease progression, we looked at expression changes in genes that might be involved with PR death in the three canine models. To this end, we used quantitative real-time PCR (qRT-PCR) with a custom-made canine specific profiling array [18] or single gene assays and examined the retinal expression of several genes that are related to vision or belong to the best described pro-apoptotic (either mitochondria-related or not), pro-survival (as down-regulation may indirectly cause cell death), and autophagy pathways. 

The complete list of genes analyzed in this study with the corresponding descriptions is reported in Table S1. Expression profiles were tested at the most relevant disease-related phases of PR cell death [19,20]: before cell death peak (*induction*; 3 wks); at cell death peak (*execution*; 5 and 7 wks); during sustained but reduced cell death rate (*chronic cell death*; > 14 wks). Our results indicate that in all three diseases, the majority of differentially expressed (DE) genes belong to the TNF superfamily and the extrinsic apoptotic pathway, and that several of them are produced by non-PR cells. Moreover, as changes in the expression of several genes were common between the three diseases, our results suggest that although different genes/mutations initiate the diseases, there may be a commonality in the signaling pathways that eventually lead to PR death.

## Results

### Morphological changes and time course of PR cell death in early-onset retinal degeneration models

**Figure 1:**
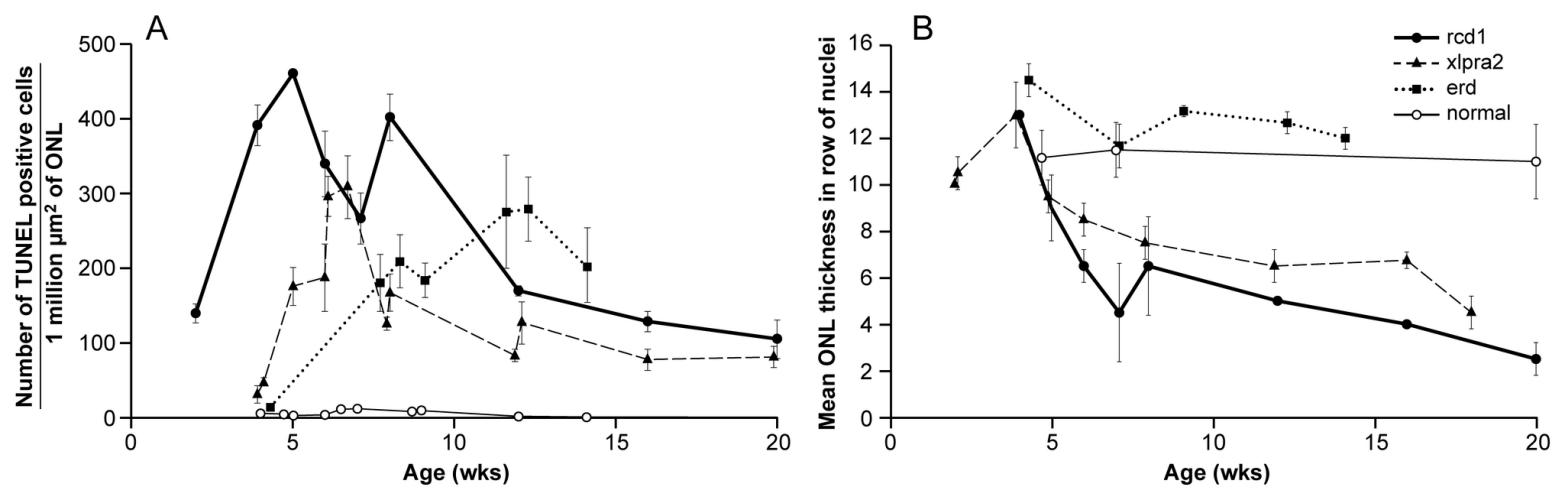
Time course of cell death in study models. **A**) TUNEL-positive PR cells as a function of age (wks) in the superior retinal meridian of normal, rcd1, xlpra2, and erd-mutants. Mean ± SD of 3 measurements taken from the superior retinal meridian of each dog are reported. **B**) ONL thickness as number of rows of PR nuclei in normal, rcd1, xlpra2, and erd-mutants as a function of age (wks). Mean ± SD of 3 measurements taken from the central and midperipheral regions of the superior retinal meridian of each dog. xlpra2 data are slightly modified from [19] and erd from [20].

As previously reported for rcd1, xlpra2, and erd, retinal development is initially normal, but PR abnormalities and retinal degeneration begin at different time points [19,21,22]; these differences are illustrated in Figure S1. We used TUNEL assays to determine the kinetics of PR cell death in rcd1, and compared the results to previous studies in xlpra2 [19] and erd [20]. While the normal retina shows only background TUNEL positive cells between 4-14 wks of age, the rcd1 retina already has a high number of dying cells at 2 wks of age, well before there is thinning of the outer nuclear layer (ONL) (Figure 1A, B). The peak of PR cell death occurs at ~5 wks, and sustained numbers of TUNEL positive cells are present from 5-7.7 wks. Although the number of TUNEL positive cells declines thereafter, it remains 100-150 fold higher than background through the 20 wks time period. 

Decreases in ONL thickness, an indication of PR loss, follow a comparable course to the apoptotic events. It occurs more rapidly and aggressively in rcd1 and slightly delayed in xlpra2. However, in erd the ONL thickness is preserved until at least 14.1 wks due to concurrent PR proliferation which is concomitant with apoptotic events [20]. At the time points examined, inner retinal neurons and Müller glial cells were morphologically normal.

### Gene and protein expression changes with disease: PR and retinal enriched genes

**Figure 2:**
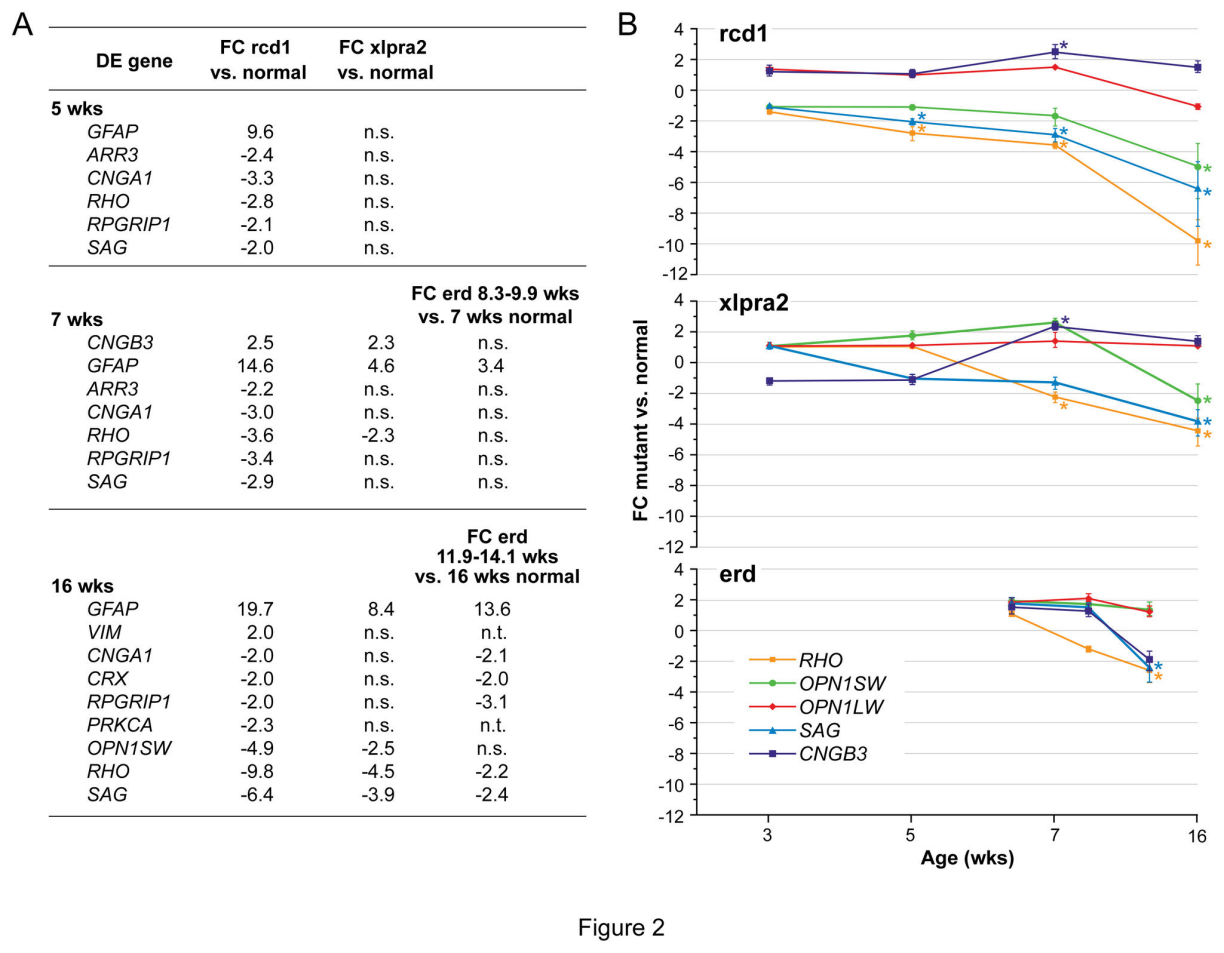
RNA expression changes of retinal genes in study models. **A**) Differentially expressed (DE) retinal genes in rcd1, xlpra2, and erd-mutants compared to normals at 5, 7, and 16 wks. No differences were found at 3 wks. DE genes are listed in alphabetical order, first the up-regulated and then the down-regulated, and are reported with the fold change (FC) differences. Note that in erd at 11.9-14.1 wks a reduced number of genes was tested (see Material and Methods). The complete list of tested genes is in Table S1. n.s. = not statistically significant differences; n.t. = not tested. **B**) FC differences between rcd1, xlpra2, and erd compared to normals at different ages (3, 5, 7, 16 wks) for *RHO*, *OPN1SW*, *OPN1LW*, *SAG*, and *CNGB3*. An asterisk indicates statistical significance, bars show SD of biological triplicates.

To assess the status of the retina, we first examined the expression profiles of PR-specific genes as well as other genes preferentially expressed in the retina. Comparison of mutant vs. normal did not show any expression changes at 3 wks of age, but there were a number of DE genes at older ages (Figure 2A). With the exception of *CNGB3* at 7 wks in rcd1 and xlpra2, only glial fibrillary acidic protein (GFAP) and vimentin (*VIM*) were up-regulated. As these genes are preferentially expressed in Müller cells, such expression is likely an inner retinal stress response to outer retinal disease. The highest numbers of DE genes, all down-regulated, were found in rcd1, reinforcing the fact that this disease is earlier in onset and more aggressive. The fold changes (FC) differences with normal of 5 selected genes, rhodopsin (*RHO*), S-opsin (*OPN1SW*), L-opsin (*OPN1LW*), S-antigen (SAG), and cyclic nucleotide gated channel beta 3 (*CNGB3*) as a function of time are shown in Figure 2B. The results for the first 4 genes illustrate the general down-regulation in expression in the three diseases with rcd1 showing a greater decrease in *RHO* and *SAG* early, but no significant changes in cone opsins until 16 wks, and then limited to *OPN1SW*. On the other hand, the cone-specific gene *CNGB3* was up-regulated in rcd1 and xlpra2 at 7 wks, but did not vary at the other ages (Figure 2B), suggesting that the expression of this gene is not correlated with the observed loss of cones. 

**Figure 3:**
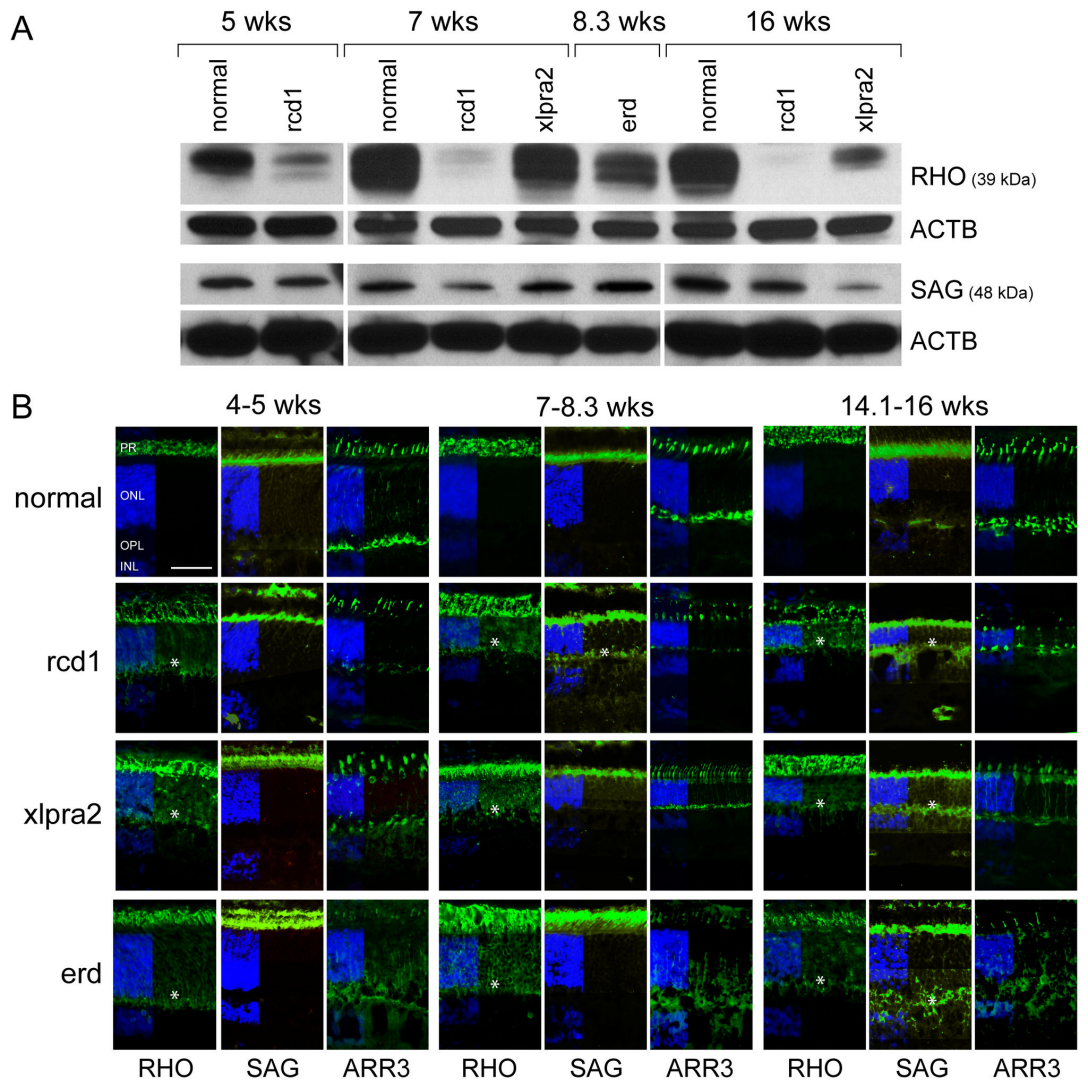
Protein expression changes of RHO, SAG, and ARR3 in study models. **A**) Western blot analysis of normal, rcd1, xlpra2, and erd retinas showed decreased levels of the rod-specific protein RHO in the tested mutants at 5, 7, and 16 wks. Decreased levels compared to normals were also observed for SAG, a major protein of the retinal rod outer segments in rcd1 at 5, 7, and 16 wks and in xlpra2 at 16 wks. The quantification of the bands illustrated in the Figure is reported in Table S2. B) Immunolabeling of normal (4, 7, 16 wks), rcd1 (5, 7, 16 wks), xlpra2 (5, 7, 16 wks), and erd (4.3, 8.3, 14.1 wks) retinas with antibodies against RHO, the cone-specific ARR3, and SAG. An asterisk denotes mislocalization of RHO and SAG in the inner retina of mutants. Note that the findings are representative for the entire retina. Scale bar: 20 μm; PR = photoreceptors; ONL = outer nuclear layer; OPL = outer plexiform layer; INL = inner nuclear layer.

To correlate gene expression changes at the mRNA and protein levels, western analysis was undertaken for selected retinal proteins. There was decreased RHO levels in rcd1 from the 5 wk time point which decreased in parallel with PR cell loss; for xlpra2 and erd, decreases occurred later. Decreases in SAG levels were more modest and occurred at all ages in rcd1 and at 16 wks in xlpra2-mutant retinas (Figure 3A and western blot quantification in Table S2). Immunolabeling results showed marked mislocalization of RHO from the outer segments to the ONL and outer plexiform layer (OPL) at all time points, and of SAG at later time points. An antibody directed against cone-specific arrestin (ARR3) was used to label the entire cone structure, and confirmed that cone cells were present, although structurally altered, in the three diseases (Figure 3B). Cell body labeling with ARR3 was present in xlpra2-mutants at early disease time points (4-5 wks) and persisted during the course of disease (Figure 3B). Similar findings have been observed in normal dogs and other mutants as well [23,24]. ARR3 labeling in erd-mutants is specific but irregular, and likely results from the continuing proliferation of PRs which, presumably, are forming new axons/synaptic terminals [20,22].

Together, our results demonstrate that significant changes in expression of a subset of the retinal genes occur and are concomitant with the onset of PR degeneration.

### Gene and protein expression changes with disease: pathway analysis

To determine how expression profiles changed as a result of disease, we compared the mutant dogs to normal controls at different ages. The results for a select subset of genes is presented in Table 1 and showed an increased expression of genes of the TNF superfamily and/or the extrinsic apoptotic pathway (comprising ligands, receptors, regulators, caspases, and suppressors), and pro-survival factors (neurotrophins and transcription factors). The results for all the genes are included in Table S3. To further delineate gene expression profiles with disease status, we discuss below relevant changes during different phases of the disease.

**Table 1:** Differential expression of selected genes in study models.

**DE gene**	**FC rcd1 vs. normal**	**FC xlpra2 vs. normal**	
***3wks***			
*CASP8* (**C**)	2.3	n.s.	
*CD40LG* (**L**)	2.6	n.s.	
*FAS* (**RE**)	2.2	n.s.	
*TNFA* (**L**)	3.5	n.s.	
***5wks***			
*CASP8* (**C**)	2.1	n.s.	
*FAS* (**RE**)	2.6	n.s.	
*NTF3* (**N**)	6.1	n.s.	
*STAT3* (**T**)	3.4	n.s.	
*TNFA* (**L**)	2.6	n.s.	
*TNFRSF1A* (**RE**)	7.7	n.s.	
*XIAP* (**S**)	-2.4	n.s.	
***7wks***			**FC erd (8.3-9.9 wks) vs. 7 wks normal**
*BDNF* (**N**)	3.1	n.s.	n.s.
*CASP3* (**C**)	n.s.	2.1	n.s.
*CASP8* (**C**)	2.8	2.1	n.s.
*CD40LG* (**L**)	30.9	15.1	3.8
*FADD* (**RG**)	2.7	n.s.	n.s.
*FAS* (**RE**)	2.9	n.s.	n.s.
*FASLG* (**L**)	4.5	n.s.	n.s.
*NGF* (**N**)	3.5	n.s.	n.s.
*NTF3* (**N**)	32.1	2.7	n.s.
*NTF4* (**N**)	4.9	n.s.	n.s.
*STAT3* (**T**)	5.1	2.4	3.7
*TNFA* (**L**)	11.2	4.3	5.3
*TNFRSF1A* (**RE**)	26.3	9.3	9.1
*TNFRSF9* (**RE**)	5.1	4.0	3.3
*TNFRSF25* (**RE**)	2.8	n.s.	n.s.
*TNFSF8* (**L**)	14.5	9.2	n.s.
*TRADD* (**RG**)	2.6	2.9	n.s.
*TRAF3* (**RG**)	2.0	n.s.	n.s.
*XIAP* (**S**)	-2.0	n.s.	n.s.
***16wks***			**FC erd (11.9-14.1 wks) vs. 16 wks normal**
*BDNF* (**N**)	2.0	n.s.	n.s.
*CASP3* (**C**)	2.1	2.0	n.s.
*CASP8* (**C**)	8.2	4.3	n.s.
*CD40* (**RE**)	2.3	n.s.	n.s.
*CD40LG* (**L**)	8.2	11.8	4.0
*FADD* (**RG**)	3.6	2.4	n.s.
*FAS* (**RE**)	8.8	3.2	n.s.
*FASLG* (**L**)	7.5	5.0	n.s.
*NFKB1* (**T**)	3.4	2.0	n.s.
*NGF* (**N**)	3.5	2.6	n.s.
*NTF3* (**N**)	42.2	48.8	n.s.
*NTF4* (**N**)	4.9	n.s.	n.s.
*RIPK1* (**RG**)	3.4	2.6	n.s.
*RIPK3* (**RG**)	9.5	3.2	n.s.
*STAT3* (**T**)	4.7	4.3	3.3
*TNFA* (**L**)	25.0	9.5	5.9
*TNFRSF1A* (**RE**)	7.3	8.8	4.1
*TNFRSF9* (**RE**)	3.2	3.9	4.1
*TNFRSF25* (**RE**)	2.9	2.9	n.s.
*TNFSF8* (**L**)	16.2	9.0	n.s.
*TNFSF10* (**L**)	9.3	4.5	n.s.
*TRADD* (**RG**)	3.2	3.7	n.s.
*TRAF3* (**RG**)	n.s.	2.2	n.s.
*XIAP* (**S**)	-2.3	-2.0	n.s.

Selected DE genes identified by qRT-PCR in rcd1, xlpra2, and erd-mutants compared to normals at 3, 5, 7, and 16 wks of age. DE genes belong to the TNF superfamily and/or extrinsic apoptotic pathway [ligands (**L**), receptors (**RE**), regulators (RG), caspases (**C**), suppressors (**S**)], are pro-survival neurotrophins (**N**) or transcription factors (**T**). They are listed in alphabetical order and are reported with the FC differences. n.s. = not statistically significant differences. Note that in erd at 11.9-14.1 wks a reduced number of genes were tested (see Material and Methods). The complete list of tested genes is available as Table S1.

#### Induction and execution phases

Based on the early onset of retinal degeneration in rcd1, we expected to see an increase in death-associated genes. In support of this observation, 8 genes were DE by the time cell death was initiated in rcd1 (3 wks), and all were up-regulated (Table S3). Four of these were members of the TNF superfamily and/or the extrinsic apoptotic pathway (Table 1). At 5 wks, the peak of cell death, 12 genes were DE (10 up- and 2 down-regulated) in rcd1 relative to normals (Table S3). These included 5 TNF superfamily and/or extrinsic apoptotic pathway members and 2 pro-survival factors; 3 of these genes were already up-regulated at 3 wks (Table 1). Down-regulated were two pro-survival genes: *XIAP*, a potent inhibitor of the extrinsic apoptotic pathway and *PRDX3*, a mitochondrial antioxidant protein that regulates NFkB. At 7 wks, there were 39 up-regulated genes, and only *XIAP* (Table 1) and *SLC25A5* were decreased in expression (Table S3). In contrast to the results of PR/retinal genes, DE genes were already apparent in rcd1 at 3 wks, indicating that changes in gene expression precede the main morphological retinal changes and suggesting that at least some of the pathways driving degeneration are already engaged at this point.

In accordance with the later disease onset and less severe PR structural abnormalities in xlpra2, there were no changes in gene expression relative to normal retinas at the 3 and 5 wk time points, which was in agreement with the PR/retinal gene expression data (Figure 2 and Table S3). However at 7 wks, 18 genes were up-regulated, and 7 of these genes were also up-regulated at the 5 wk time period in rcd1. A comparison of the 18 DE genes in xlpra2 at 7 wks with rcd1 at the same age shows that 17 of 18 genes were identical and showed the same pattern of regulation. The only exception was *CASP3*, which was found to be up-regulated only in xlpra2. These data further suggest that although rcd1 and xlpra2 are caused by different mutations, the changes in gene expression profiles are similar, with only small differences in the kinetics of expression.

A smaller number of time points were available for erd. No differences in expression were found between 6.4 wks old mutants and 7 wks normals. However, at 8.3-9.9 wks, 6 genes (*CD40LG*, *HSP90*, *STAT3*, *TNFA*, *TNFRSF1A*, *TNFRSF9*) were up-regulated, and these were also DE in rcd1 and xlpra2 (Table S3). Of these, two are TNF superfamily ligands, two TNF superfamily receptors, and one a pro-survival transcription factor (Table 1). These findings suggest that similar mechanisms may be involved in triggering the cell death cascade in all three diseases. Moreover, the higher number of DE genes found in rcd1 relative to both other diseases and the relative kinetics of cell death support the observations that rcd1 is the more aggressive disease. The limited expression changes observed in erd indicate that cell death is not the major disease feature at this age.

#### Chronic cell death phase

This disease phase is characterized by a lower rate but continuous cell death process, particularly in rcd1 and xlpra2, which is associated with a similar pattern of decreased ONL thickness. We identified many DE genes in signaling pathways in the three mutants at this phase, most of which were up-regulated: 42 in rcd1, 37 in xlpra2, and 8 in erd (Table S3). As at the 7 wk time point, all up-regulated genes in erd were also up-regulated in the other two diseases. *XIAP* was down-regulated in both rcd1 and xlpra2, and *SLC25A5* was also down-regulated in the latter (Table S3). A total of 15 genes belonging to the selected functional groups were DE in both rcd1 and xlpra2, but not erd (note that a smaller number of genes were evaluated in erd at this age; see Material and Methods), while 3 and 1 were rcd1- or xlpra2-specific, respectively (Table 1). 

When comparing the DE genes at 16 wks relative to 7 wks, we identified several common gene expression signatures. Specifically, up-regulation of 5 genes that belong to either the TNF superfamily, the extrinsic apoptotic pathway, or are a pro-survival transcription factor (*CD40LG*, *STAT3*, *TNFA*, *TNFRSF1A*, *TNFRSF9*) was found in all three diseases at both time points (Table 1). Additionally, a number of genes that belong to the same functional groups (*CASP8*, *NTF3*, *TNFSF8*, *TRADD*) were up-regulated in rcd1 and xlpra2, but not erd, at both ages (Table 1). These findings show that, although the three diseases are non-allelic, common signaling pathways are shared among the three and extend from the *execution* phase to the *chronic cell death* phase.

#### Fold change differences for selected genes belonging to different gene categories

**Figure 4:**
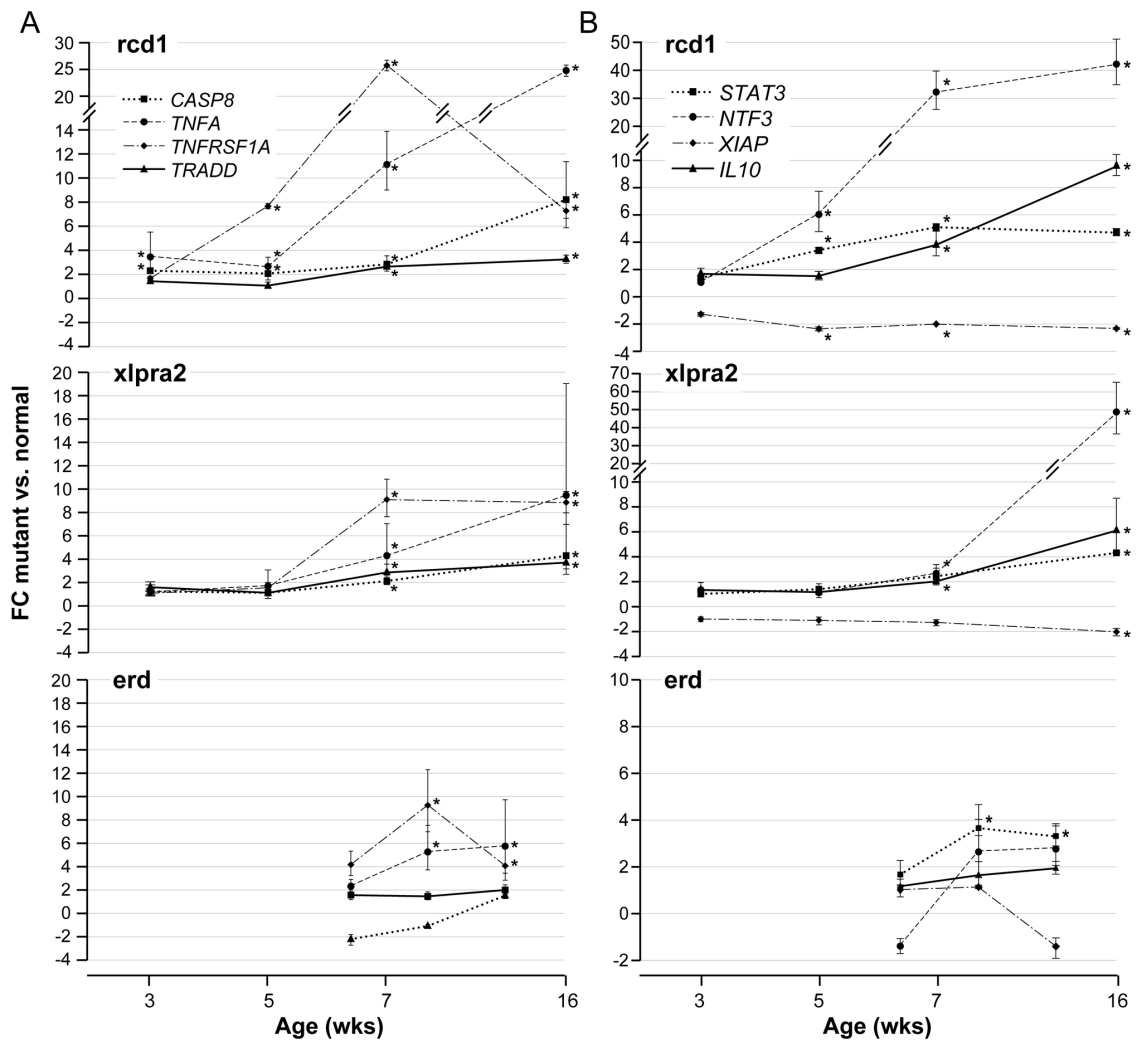
RNA expression changes of selected pro-death and pro-survival genes in study models. The fold change (FC) differences measured by qRT-PCR of selected genes in mutants compared to normals are shown at different ages. Genes belong to different functional categories: **A**) pro-death members of the extrinsic apoptotic pathway *CASP8*, *TNFA*, *TNFRSF1A*, *TRADD*; **B**) pro-survival *STAT3*, *NTF3*, *XIAP*, *IL10*. An asterisk indicates statistical significance; bars show SD of biological triplicates. Note that values on the Y-axis are not the same for all graphs, due to the highly variable FC differences in gene expression.

The profiling array results highlighted a potential role in the degeneration process for genes of the extrinsic apoptotic pathway and those having a pro-survival function. To better illustrate these results we displayed FC differences compared to normals at different ages for selected genes representing these functional categories (Figure 4A: pro-death members of the extrinsic apoptotic pathway; 4B: pro-survival genes). With the exception of *XIAP*, there is an increased expression of all these genes. The magnitude and time course of expression directly reflected the severity and rate of progression of the diseases, being earlier and of greater magnitude in rcd1. These data suggest that although apoptotic pathways are activated during the disease process, counteracting pro-survival mechanisms are also engaged, albeit to a lesser extent.

#### Protein analysis of selected DE genes

To assess the differential RNA expression results of the pathway analysis at the protein level, DE genes representing different functional groups that were differentially regulated in diseased dogs were selected for analysis by western blot and/or IHC at relevant time points. These included TNF superfamily members and/or members of the extrinsic apoptotic pathway, as well as pro-survival genes.

#### Pro-death members of the extrinsic apoptotic pathway are up-regulated during early PR degeneration

**Figure 5:**
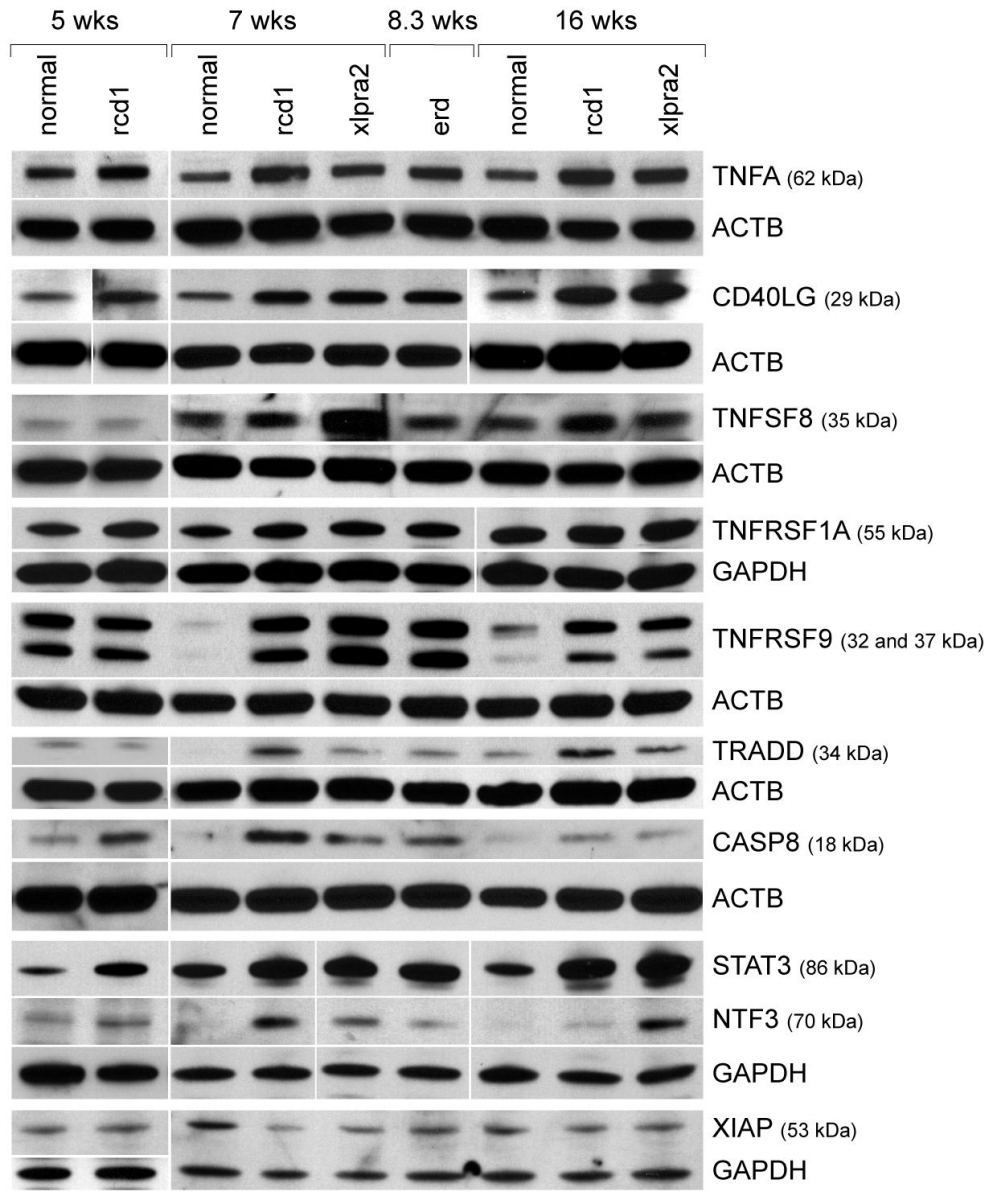
Protein quantification by western blot in retinas of study models. Protein expression of TNF superfamily ligands (TNFA, CD40LG, TNFSF8), TNF superfamily receptors (TNFRSF1A, TNFRSF9), TNF superfamily regulator TRADD, initiator caspase CASP8, and pro-survival molecules STAT3, NTF3, and XIAP were analyzed at different ages of normal, rcd1, xlpra2, and erd retinas. Up-regulation of TNFA and CD40LG was found in mutants at all ages, particularly at 7-8.3 and 16 wks. TNFSF8 expression was markedly higher in xlpra2 at 7 wks and rcd1 at 16 wks. TNFRSF1A was marginally increased in mutants at all ages, while TNFRSF9, TRADD, and active CASP8 were up-regulated at 7-8.3 and 16 wks. Active CASP8 was also increased in rcd1 at 5 wks. Both STAT3 and NTF3 were up-regulated in mutants at all ages, whereas XIAP expression decreased in mutants after 7 wks, and was particularly low in rcd1 at 7 wks. Either ACTB or GAPDH were used as loading controls. White spaces indicate that the gel was cut. Approximate molecular size markers are indicated. The quantification of the bands illustrated in the Figure is reported in Table S2.

**Figure 6:**
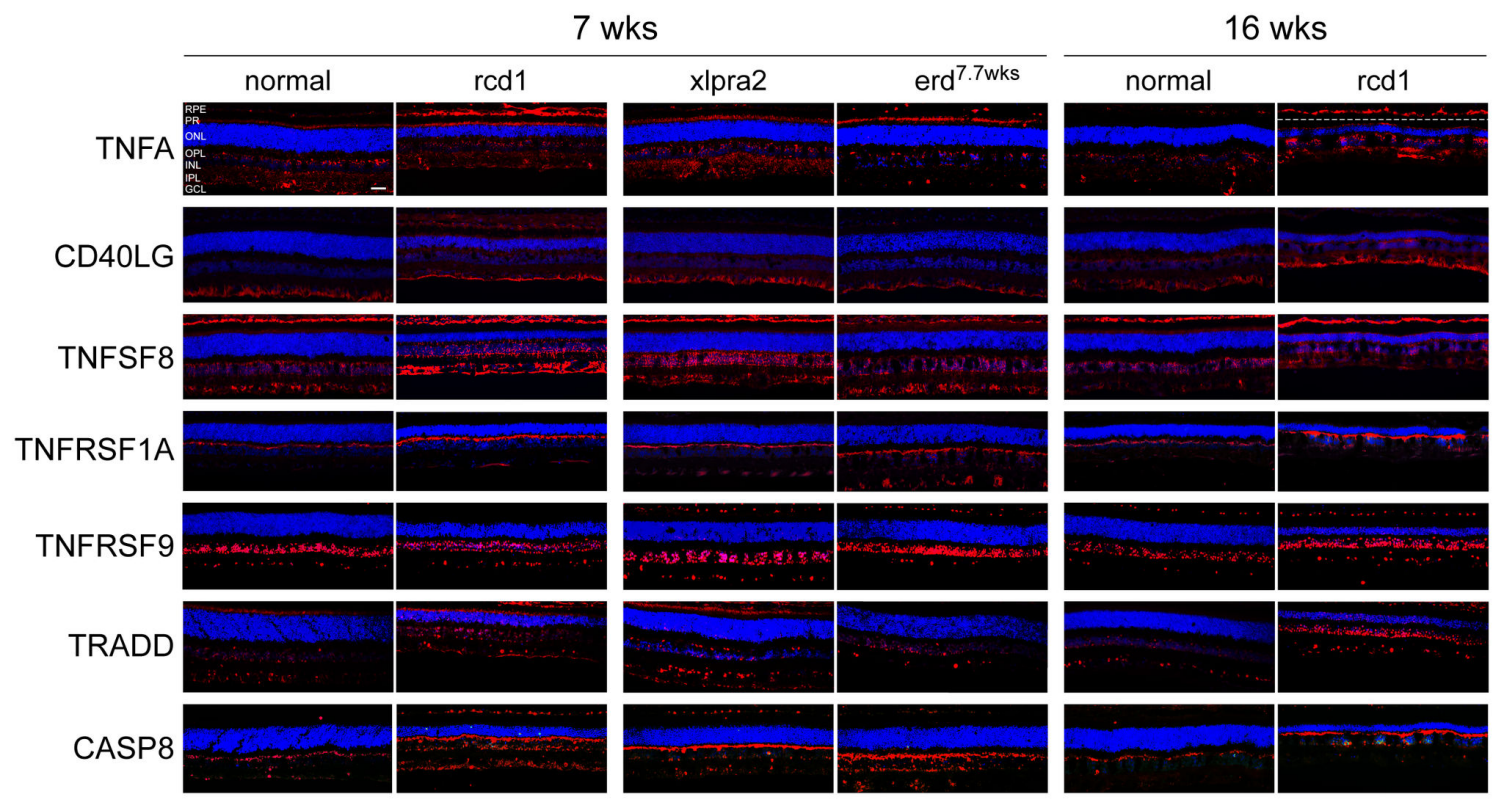
Retinal localization of proteins of the TNF superfamily and CASP8 in study models. Immunolabeling of normal, rcd1, xlpra2, and erd retinas with antibodies against TNF superfamily ligands (TNFA, CD40LG, TNFSF8); TNF superfamily receptors (TNFRSF1A, TNFRSF9; TNF superfamily regulator TRADD; and the initiator caspase CASP8. TNFA was localized to the inner retina in Müller cells (Figure S2) at all ages. In mutants, labeling was more intense and present in RPE and remaining cone inner segments. CD40LG and TNFSF8 expressions were comparable to normals, although levels were higher in mutants, particularly at 16 wks in rcd1. Both proteins were found in the inner retina, CD40LG mainly in the GCL and Müller cells (see Figure S2), while TNFSF8 was more widely distributed and co-localized with Müller, horizontal, and ON bipolar cells (see Figure S2). TNFRSF1A localized mainly to the OPL in horizontal cells (see Figure S2). The staining intensity was higher in mutants than in normals, particularly in erd at 7.7 wks and rcd1 at 7 and 16 wks. TNFRSF9 and TRADD labeling was comparable in normal and mutants, although both were slightly more intense in the latter. They both were localized to the OPL and INL, where they co-localized with horizontal cells and TNFRSF9 also with amacrine cells (see Figure S2). TNFRSF9 was present in the majority of INL cell nuclei, while TRADD expression was limited to fewer cells. TRADD labeling was also present transiently in PRs at 7 wks. CASP8 mainly localized to the OPL in horizontal cells (see Figure S2), and the labeling intensity was higher in mutants compared to normals. Scale bar: 20 μm; RPE = retinal pigment epithelium, PR = photoreceptors, ONL = outer nuclear layer, OPL = outer plexiform layer, INL = inner nuclear layer, IPL = inner plexiform layer, GCL = ganglion cell layer, NFL = nerve fiber layer.

We initially tested protein expression of TNF superfamily ligands TNFA, CD40LG, and TNFSF8. TNFA was slightly up-regulated in rcd1 at 5 wks and in the three mutant retinas at 7-8.3 wks. At 16 wks, samples were only available for rcd1 and xlpra2 and TNFA levels were elevated in both (Figure 5, Table S2). A similar expression pattern was observed for CD40LG and TNFSF8, although the latter was markedly higher at 7 wks in xlpra2 and at 16 wks in rcd1, but did not vary at 8.3 wks in erd (Figure 5, Table S2). Immunohistochemistry (IHC) was used to identify the cells and retinal layers in which the protein expression changes occurred. Indeed, to specifically identify positive cells, dual-labeling of selected proteins with either Go-α (marker for ON bipolar cells), parvalbumin (marker for horizontal and amacrine cells), or CRALBP (marker for Müller cells) was performed. In normal retinas, TNFA was localized to the RPE only in young dogs at 5 wks (data not shown), to the PR layer at 7 wks, and to the inner retina, in particular to Müller cell end feet (Figure S2), at all ages examined. This distribution pattern was also present in rcd1, xlpra2, and erd but labeling was more intense and expression persisted in the RPE and remaining cone inner segments at all ages (Figure 6). Localization of CD40LG and TNFSF8 in mutant retinas was comparable to normal, although expression levels were higher (Figure 6). Both proteins were found in the inner retina, CD40LG mainly in the GCL, where it co-localized with Müller cell end feet (Figure S2), while TNFSF8 was observed in all layers, and co-localized with Müller, horizontal, and ON bipolar cells (Figure S2). Interestingly, the retraction of ON bipolar dendrites previously reported in xlpra2 compared to normals [25] is clearly visible with Go-α (Figure S2). These data show that while all 3 TNF superfamily ligands are present in Müller cells, TNFSF8 also appears to localize in other inner retinal neurons.

We next examined the expression of the TNF superfamily receptors TNFRSF1A and TNFRSF9, and the interacting regulator TRADD. Western analysis showed that while expression of TNFRSF1A was marginally increased in the three mutants, TNFRSF9 and TRADD were up-regulated in the *execution* and *chronic cell death* phases (Figure 5, Table S2). By IHC, the localization of these proteins was comparable to normal, and increased labeling was found in erd at 7.7 wks and rcd1 at 16 wks. IHC localized TNFRSF1A mainly to the OPL in all tested retinas and to a lesser extent to the inner retina. In normals at 7 and 16 wks, the staining intensity was lower than in the age-matched mutants, in particular the 7.7 wks erd and the 7 and 16 wks rcd1 (Figure 6). Confocal microscopy showed that TNFRSF1A co-localized with the horizontal cell lateral extensions in both normal and mutants (Figure S2), while the protein was not present in ON bipolar cells (data not shown). Both TNFRSF9 and TRADD, a regulatory protein that can bind to TNFRSF1A, were localized to the INL, and to a lesser extent, the GCL and NFL (only TRADD). In the INL, TNFRSF9 was present in all cell nuclei, while TRADD expression was more limited and fewer cells were labeled (Figure 6). Both proteins were co-localized in the OPL to few horizontal cells, and TNFRSF9 also to amacrine cells (Figure S2). Both proteins were not expressed by ON bipolar and Müller cells (data not shown). These results indicate that the same cells express the 2 receptors and the adaptor molecule, but at this point, their involvement in the PR degenerative process is unknown.

The initiator and effector caspases, CASP8 and CASP3 respectively are activated during the extrinsic apoptotic pathway, and our mRNA data showed an increased expression of the genes (Table 1). Western blot results showed that the 18 kDa active form of CASP8 was up-regulated in rcd1 at 5 wks, and in mutants at 7-8.3 and 16 wks (Figure 5, Table S2), while we could not identify the non-active form of the protein (65 kDa). IHC results showed that labeling intensity of active CASP8 was higher in mutants compared to normal at 7 and 16 wks and that the protein mainly localized to the OPL (Figure 6) in horizontal (Figure S2) but not ON bipolar cells (data not shown). In contrast, western and IHC failed to identify the specific active form of CASP3 (17 kDa) in our retinal samples (data not shown) despite testing 5 different antibodies (Table S4). Thus, our current data does not confirm nor reject the hypothesis of caspase-dependent cell death in these canine models.

#### Pro-survival responses are both up- and down-regulated during early PR cell death

**Figure 7:**
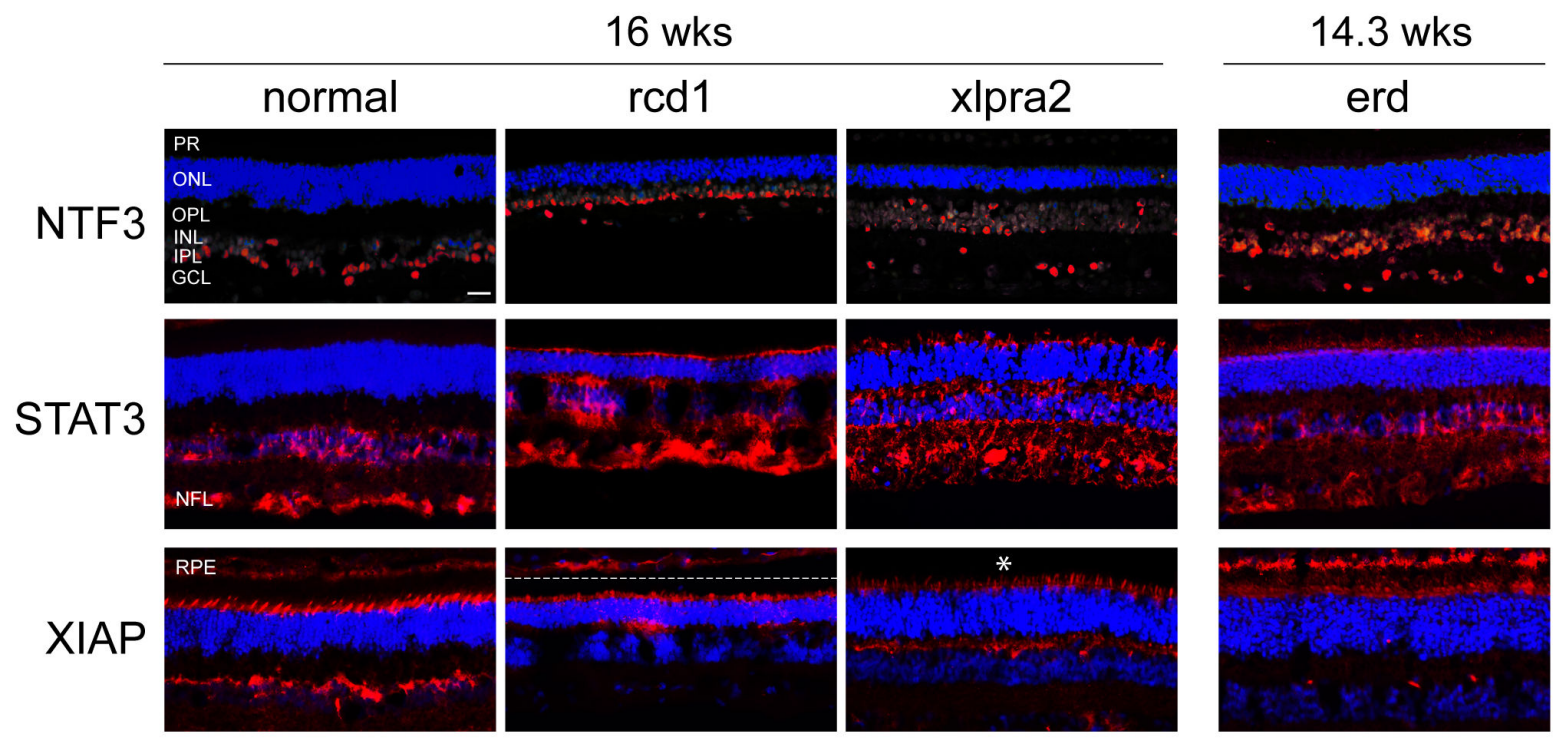
Retinal localization of pro-survival proteins in study models. Immunolabeling of normal, rcd1, xlpra2, and erd retinas at 14.3 to 16 wks with antibodies against pro-survival proteins STAT3, NTF3, and XIAP. Both STAT3 and NTF3 were primarily localized to the inner retina, from the INL to NFL, although the labeled cells differed. Mutant retinas exhibited higher labeling intensities and STAT3, but not NTF3, and showed intense PR inner segment labeling that was absent in normals. STAT3 also co-localized with Müller cells (see Figure S2). Mutant and normal retinas showed similar XIAP labeling pattern, although the intensity was reduced in mutants. XIAP was found in the PR layer, including the OPL-INL interface, as well as the RPE. PR-labeling was restricted to IS that in terms of numbers and shape appeared to be cones. An asterisk indicates that the RPE was missing. Scale bar: 20 μm; RPE = retinal pigment epithelium, PR = photoreceptors, ONL = outer nuclear layer, OPL = outer plexiform layer, INL = inner nuclear layer, IPL = inner plexiform layer, GCL = ganglion cell layer, NFL = nerve fiber layer.

We examined the expression of 3 pro-survival/anti-apoptotic proteins that were DE at the RNA level, as well as expression of the canonical NFkB pathway which is downstream of TNFRSF1A. In accordance with the RNA expression results, higher STAT3 and NTF3 protein levels were found in mutants (Figure 5, Table S2). IHC results indicated that, at 16 wks, the transcription factor STAT3 and the neurotrophin NTF3 were primarily localized from the INL to NFL of the inner retina, although the specific cell labeling pattern differed (Figure 7). Labeling was similar in normals and mutants although the latter showed higher intensity. In addition, STAT3 localized to the soma and Müller cell end feet (Figure S2) and, unlike NTF3, showed intense labeling of PR inner segments at the later time point, a finding not observed in normals (Figure 7). 

On the other hand, western analysis indicated that the expression of the anti-apoptotic XIAP decreased in rcd1 and xlpra2 mutants beginning at 7 wks, with particularly low levels in rcd1 (Figure 5, Table S2). In normals at 16 wks, IHC showed intense XIAP staining in the PR layer, the OPL-INL interface, as well as the RPE. PR-labeling was restricted to the IS, and was not generalized to all cells (Figure 7). The same staining pattern was also observed at earlier ages, i.e. 5-8 wks (data not shown). By the cytological appearance and distribution, the XIAP positive IS appeared to be cones. Dual-labeling with monoclonal RHO and polyclonal XIAP antibodies confirmed that the XIAP positive cells were not rods as no co-localization was observed (data not shown). Mutant retinas showed a similar labeling pattern as normals, particularly in xlpra2, although the intensity was much reduced. Thus, the observed down-regulation of this anti-apoptotic protein in cones may contribute to the secondary loss of this cell class in rod or rod-cone degenerations.

As the TNFA-TNFRSF1A interaction is known to lead to the activation of the pro-survival NFkB pathway [26], we examined the phosphorylated state of 3 key signaling molecules NFkB p65, IKKα/β, and IKBα. Although these proteins were activated in positive controls from dogs with relapsed B-cell lymphoma, western blot analysis did not identify any phosphorylation of these proteins in retinal samples. The antibodies also did not label any normal or mutant retinal cells by IHC, despite labeling canine tonsil tissues used as positive controls. These findings suggest that the canonical NFkB pathway is not activated during retinal degeneration and it is not the downstream pathway activated by TNFA in the three tested models.

## Discussion

### Relevance of the study

This study examined the expression profiles of genes in pathways relevant to PR degeneration in canine early-onset models with the goal of identifying potential pathways and genes that would be involved in the progression from mutation to PR cell death. Differential expression results were consistent at both the RNA and protein levels, and pointed to genes and pathways potentially involved in the disease. As transcriptome profiling arrays require validation of a subset of DE genes by qRT-PCR, we decided instead to develop a qRT-PCR array to directly examine the expression of selected genes in pathways [18]. For this analysis, time points were selected based on cell death kinetics (Figure 1 and [19], [20]) and covered temporally distinct phases of the disease-*induction*, *execution*, *chronic cell death*-that are readily analyzed with minimal overlap.

Comparison between mutants vs. normals showed two different expression profile patterns. Mutant retinas up-regulate expression of several genes of the TNF superfamily, the extrinsic apoptotic pathways, and pro-survival neurotrophins and transcription factors, while they down-regulate PR-specific genes. Only Müller cell *GFAP* expression, which reflects an inner retinal stress response to outer retinal disease in several retinal diseases including xlpra2 [27], was markedly elevated at early time points. Other than the mRNA and protein expression changes occurring in Müller cells, these cells showed no structural changes during the time points of the disease examined.

Although the genes and pattern of expression are similar between diseases, the timing is not, and is dependent on the temporal kinetics of cell death. When these are aligned, then the similarities are striking, particularly for rcd1 and xlpra2 that have a narrower *execution* phase. The delay in expression changes in erd until 14.1 wks supports a slower disease time course, and is consistent with the sustained apoptosis and cell division that occurs during this time period [20].

Decreased expression of *CRX*, *RHO*, *OPN1SW*, and *SAG* has been previously reported in xlpra2 at 16 wks [27], and changes in PR-specific gene expression, such as down-regulation and mislocalization of RHO to the ONL and OPL, are in agreement with studies in mice [28,29] and dogs [19,20]. These findings provide added support that the mutant PRs are compromised. Moreover, ARR3 expression data confirmed that cone abnormalities occurred at later ages, after a substantial damage to rods occurred, even though no changes were observed in *OPN1LW* expression. These findings indicate that in all three diseases a differential and preferential damage of rods and S-cones occurs, which is dependent on the time course and severity of the disease. 

In order to investigate the effects of the mutations on disease onset and progression, we examined for the possible activation of intrinsic and/or extrinsic pathways of cell death in the retina. It is possible that the approach used may have missed genes not included in the array, or molecular pathways that involve post-translational activation and not expression changes at the transcript level. In spite of these limitations, our results identified alterations in the expression of several genes and proteins of the TNF superfamily and the extrinsic apoptotic pathway (for reviews see [30,31]), and double-immunolabeling techniques suggest that some of these proteins are produced by Müller and horizontal cells. 

In mutant retinas we observed increased expression of the three TNF superfamily ligands, TNFA, CD40LG, and TNFSF8, which were expressed by Müller glia and other retinal cells. Activation of Müller cells is a hallmark of early PR disease, and represents a stress response indicating early signaling events from the outer retina to the Müller cells that may play a role during the degeneration process. Such reactivity of glial cells secondary to neuronal injury is also observed in human glaucomatous eyes [32] and retinal ischemia in pigs [33]. In addition to Müller cells, intense TNFA labeling was found in mutant retinas, particularly in RPE and remaining PRs. However, the principal TNFA receptor, TNFRSF1A, showed minimal changes at the protein level, was localized to horizontal cells as previously reported [33]. Thus, it is not clear at this time if TNFA transmits signals to PRs indirectly through inner retinal neurons, or directly through a receptor yet to be identified. Alternatively, binding of TNFA to TNFRSF1A could lead to activation of the canonical pro-survival NFkB pathway (reviewed by [26]), but this was not supported by our finding of lack of phosphorylation of NFkB p65, IkBα, and IKKα/β.

In glaucomatous neurodegeneration, TNFA has been suggested to induce apoptosis via TNFRSF1A and through the extrinsic apoptotic pathway by activation of CASP8 and CASP3 [34]. This process has been shown to be co-regulated by the adaptor protein TRADD [31]. In addition to TNFA and TNFRSF1A, we found increased RNA expression of CASP8, CASP3, and TRADD. Indeed, elevated protein expression of active CASP8 and TRADD could be confirmed by western blot analysis, and both proteins, in addition to TNFRSF1A, were expressed by horizontal cells. These results suggest that these cells may play a relevant role in the disease mechanism. However the specific interactions between these proteins and PR degeneration still has to be determined.

The specific role of caspases in PR apoptosis remains controversial. In models of retinitis pigmentosa, increased CASP3 activity caused PR degeneration in transgenic S334ter rats, tubby mice, rds and rd mice with concomitant activation of CASP8 and mitochondrial release of cytochrome C. In contrast, CASP3-independent apoptosis and rod death has been reported in rd mice (reviewed by [11]). In our studies, CASP8 was slightly activated in mutants, but we could not confirm CASP3 activation. TNFA has been shown to induce both apoptosis and necroptosis, an alternative form of programmed cell death that can occur when caspase-dependent apoptosis is inhibited [35]. CASP8 plays a crucial role in both; it initiates apoptosis and, in conjunction with FADD, negatively regulates RIPK1/3-mediated necroptosis [35,36]. Interestingly, we found increased *FADD*, *RIPK1*, and *RIPK3* expression in rcd1 and xlpra2 during the *chronic cell death* phase, when CASP8 protein expression (Figure 5) is decreasing. However, further analyses, including later time points, to elucidate the exact role of caspases and the necroptosis pathway in canine retinal degeneration models will be required to ascertain the specific roles of these proteins. 

We found increased TNFRSF9 expression in mutants, and the protein is expressed in cell nuclei and by a subset of horizontal and amacrine cells. While TNFA-induced TNFRSF9 expression contributes to activation of regulatory T cells [37], and promotes a STAT3/FAS dependent signaling pathway in dendritic cells [38], the role of this receptor in the retina is not known. As expression of *FAS*, its ligand *FASLG*, and the adaptor *TRADD* were up-regulated in mutant retinas, FAS-mediated apoptosis may contribute to the degeneration process in our models. In support of this hypothesis is the finding that this apoptotic pathway, including *FAS*, *FASLG*, and *CASP8*, are activated in Brown-Norway rats after retinal detachment [39]. Moreover, the FAS pathway can also trigger a CASP8-independent cell death pathway using RIPK (up-regulated in this study) as effector molecule [40]. These data suggest that in the canine models the extrinsic apoptotic pathway and others pathways might be activated in the same or different cell populations during PR degeneration [8].

The profiling array used in the studies also interrogated genes with pro-survival functions. Of particular significance was the down-regulation of XIAP, a potent anti-apoptotic protein expressed in canine cone PRs (present study), and shown to inhibit caspases (e.g. 3,7,9), and suppress apoptosis [41]. This protein has a critical pro-survival role in the retina [42] and other tissues [43], and decreased levels of expression indicate that a compromised XIAP response may accelerate cell death. Experimental gene delivery studies with *XIAP* suggest some degree of structural and functional rescue to PRs in both acute and chronic rodent models of retinal degeneration [44]. 

PR survival can also be achieved by promoting the production of neurotrophic factors. Our studies show increased message level of NGF, NTF3, and NTF4, and we localized NTF3 to nuclei of most cells in the INL and ONL. We also tested the endogenous expression of additional neurotrophic factors, e.g. BDNF, BFGF, GDNF, CNTF, that have been shown to protect and delay neurodegeneration in animal models of RP (reviewed by [4]). Except for BFGF, whose expression did not vary, *BDNF*, *CNTF*, and *GDNF* RNA expression was slightly up-regulated in rcd1 at 7 and 16 wks, while *GDNF* was also up-regulated in xlpra2 at 16 wks. STAT3, a transcription factor with a neuroprotective role in PRs after light damage [45], was also up-regulated in mutants in this study, and was expressed not only in Müller cells, but also in mutant PR inner segments. These results further indicate that multiple pathways with opposing functions and levels of response, some pro-death and others pro-survival, are activated during the degeneration process, likely contributing to the timing and severity of the disease. 

A previous study indicated that PR-derived EDN2 functions as a general stress signal to Müller cells by binding to EDNRB [46]. In this study, slight up-regulation of EDN2 occurred without changes in EDNRB expression. This suggests that this mechanism might not be involved in the cross-talk between PRs and Müller cells in our models. 

We previously reported that a number of non-apoptotic genes related to mitochondria were altered in xlpra2-mutants [27], but the present results do not support an involvement of the apoptotic mitochondrial pathway in the degeneration process. No differences were found in the expression of the apoptogenic factors, or the BCL2-family members that are key players in the execution of the mitochondrial apoptotic pathway [11]. Although few genes of the latter family were slightly up-regulated in rcd1 (anti-apoptotic: *BCL2* at 7 wks; pro-apoptotic: *BAK1* at 7 wks and *BBC3* at 3, 7, 16 wks), their opposite role and the limitation to one disease and specific time-points indicate that they might not be relevant components of a general degenerative process. Retinal degeneration in different mouse models has been reported to occur from non-apoptotic mechanisms involving changes in cyclic nucleotide metabolism (i.e. down-regulation of CREB and up-regulation of calpains [9]), and induction of autophagy with activation of cathepsins [8,10]. In our study, we also found an age specific up-regulation of some calpains and autophagy related genes, but others, e.g. *CREB1*, *CTSD*, and *ATG3*, -6, -7, -12, did not change in any of the diseases. These results suggest that, in contrast to the mouse models, cyclic nucleotide metabolic abnormalities or the autophagy pathway are not essential mechanisms. However, before excluding them and the mitochondrial pathway as being involved in PR degeneration, additional analyses at the protein level, including the examination of post-transcriptional modifications, are needed.

Based on our results, we propose that a complex death regulatory network is present in retinal cells in which TNFA-mediated signaling pathways, horizontal and Müller cells appear to play a relevant role and may act in conjunction with multiple diverse other apoptosis and/or pro-survival promoting gene products and pathways that are yet to be defined. Other cells might also contribute to the degeneration process, as several DE expressed genes identified in this study, e.g. *CCL2*, *CD18*, *CD40LG*, *CD45*, *STAT3*, *TNFA*, *TYROBP*, have been shown to activate or be expressed by microglia, but further studies are warranted to confirm this hypothesis. The possibility that activation of TNFA-related pathways in horizontal and Müller cells is detrimental or favorable to the PR can be tested with inhibitors. Positive modulation of the disease would suggest that they have a pro-death role, while worsening of the disease would indicate a pro-survival role. These studies are ongoing.

## Materials and Methods

### Ethics statement

The research was conducted in full compliance and strict accordance with the Association for Research in Vision and Ophthalmology (ARVO) Resolution on the Use of Animals in Ophthalmic and Vision Research. The protocol (number: 801870) was approved by the University of Pennsylvania Institutional Animal Care and Use Committee (IACUC). All efforts were made to minimize dog suffering.

### Tissue samples

Age-matched normal and mutant dogs with a common genetic background were maintained at the Retinal Disease Studies Facility in Kennett Square, Pennsylvania, and retinal tissues were collected as previously reported [27]. Three different canine models were used for the studies: a) rod cone dysplasia 1 (rcdl) is an early-onset, autosomal recessive rod disease caused by a nonsense mutation in the rod-specific cyclic GMP phosphodiesterase ß subunit (*PDE6B*) that results in a stop codon and truncation of the protein by 49 aa [14,15]. The mutation in *PDE6B* results in a comparable disease to that in rd1 and rd10 mice. cGMP accumulates in mutant PRs and eventually triggers cell death via pathways that presumably involves Ca^++^ influx through CNG channels in rod OS [47]. As in our model, cone PRs, although unaffected by the mutation, also degenerate secondarily; b) X-linked progressive retinal atrophy 2 (xlpra2) is the dog homolog of X-linked retinitis pigmentosa (XLRP). It is an early-onset, rod and cone disease caused by a 2-bp microdeletion in *RPGR* exon ORF15 creating a frameshift and premature stop in the translated protein [16]. The protein localizes to the connecting cilium, and participates in intraflagellar protein transport. This ciliary protein is essential for PR viability and plays a role in ciliogenesis, however its function is not yet entirely understood (reviewed by [48]; **c**) early retinal degeneration (erd) results from a mutation in *STK38L* which appears to be involved in early PR development [17,20]. The disease is characterized by abnormal development and degeneration of rods and cones and, as an unique feature, by concurrent PR apoptosis or mitosis, and formation of hybrid rod/S-cone cells [20]. Although the function of this protein in PRs is presently unknown, recent *in vitro* studies indicated that STK38L-mediated Rabin8 phosphorylation appears to be crucial for ciliogenesis [49].

Gene expression profiles using the canine specific qRT-PCR profiling array described below were determined for age-matched 3, 5, 7, and 16 wks old normal, rcd1, and xlpra2-mutants (3 biological replicates/time point/group), as well as two erd-mutants at 6.4 wks and three at 8.3/9.9 wks of age. Single expression assays of 38 selected genes (*CD40LG*, *TRADD*, *CASP8*, *IL6*, *IL10*, *NTF3*, *PTPRC*, *EDN2*, *EDNRB*, *NFKB1*, *CRX*, *SAG*, *CNGB3*, *CNGA3*, *BNIP3L*, *PLAGL2*, *ITGB2*, *RPGRIP1*, *RPGR*, *NDUFS4*, *FADD*, *SLC25A5*, *CNGB1*, *CNGA1*, *RPGRORF15*, *GFAP*, *OPN1LW*, *OPN1SW*, *RHO*, *RIPK1*, *RIPK3*, *TNFA*, *XIAP*, *CCL2*, *STAT3*, *TNFRSF1A*, *TNFRSF9*, *ZBTB4*) were analyzed in two erd-mutants at 11.9 and 14.1 wks.

### Experimental time points

Expression profiles were tested at the most relevant disease-related phases of PR cell death [19,20]. The 3 wk time point (*induction* phase) is at the onset of disease prior to the peak of PR death, and the retina is comparable in structure to normal. The disease dependent *execution* phase at 5 and 7 wks coincides with the peak of PR cell death, and occurs during or shortly after the end of complete, albeit abnormal, postnatal retinal development. During this phase, there is outer segment disorganization and disruption, but most of the diseased cells are present suggesting that any detected alterations in gene expression are likely to represent early degenerative processes. Lastly, at > 14 wks (*chronic cell death*) the mutant retina shows sustained but reduced cell death rate and a persistent low-grade degeneration.

### TUNEL assay and ONL thickness measurement

Cryosections along the superior meridian of rcd1 mutants were analyzed with TUNEL (terminal deoxynucleotidyl transferase mediated biotinylated UTP nick end labeling) assay, as previously reported for xlpra2 [19] and erd [20]. TUNEL positive apoptotic nuclei were visualized with the In Situ Cell Death Detection kit (Roche Applied Science, Indianapolis, IN) and stained with 4′,6′-diamino-2-phenylindole (DAPI). Positive controls included sections pre-treated with DNase I (3 U/mL in 50 mM Tris-HCl [pH 7.5] and 1 mg/mL BSA). In negative controls, the terminal transferase enzyme was omitted from the TUNEL reaction mixture. Sections were examined from the optic disc to the ora serrata by epifluorescence microscopy with the 40x objective. TUNEL-labeled cells in the ONL were counted throughout the entire length of the section. The number of PRs undergoing cell death as a function of time was expressed as the number of TUNEL-labeled PRs per 10^6^ μm^2^ of ONL. The area of the ONL of each section was obtained by measuring the entire length of the ONL, from optic disc to ora serrata, and multiplying it by the average thickness (measured in three different locations) of the ONL throughout the section. Each dog was tested in triplicate with sequential sections from the superior meridian and the values were averaged and reported as the mean ± SD.

For each dog, a single section from the superior quadrant was used for quantitative evaluation of ONL thickness, measured as the number of rows of PR nuclei. Two specific locations were examined; 2,000 ± 500 µm from the optic disc and mid point ± 500 µm between optic disc and ora serrata. At each of these sites, the number of rows of PR nuclei in the ONL were counted in at least three areas of a 40 field and averaged.

### Canine specific qRT-PCR profiling array

The canine-specific TaqMan qRT-PCR profiling array was developed in conjunction with Applied Biosystems and validated on a smaller number of genes using control and staurosporin-treated MDCK cells [18]. The custom made array included genes that are present in other commercially available arrays for other species, and potentially relevant genes identified from the literature which might have a role in the studied canine retinal disorders. The array has been extensively described and the nature of the genes present on the array was analyzed with the Ingenuity Pathways Analysis (IPA) program [18]. 

Details on the updated v4 array are presented in Table S1. The Table also includes the genes analyzed by single assays using either TaqMan or SYBR green reagents. For the latter, the specificity of the primers was confirmed by dissociation curve procedures as a single melting temperature was observed which excluded the presence of secondary non-specific gene products and primer dimers. Genes in the array were sub-divided into 6 main categories that inform on signaling pathways and disease mechanisms, according to the current literature. This is somewhat arbitrary because several genes fit into different categories depending on various factors, e.g. cell type, disease, age, interaction with different other molecules, and because this classification is a dynamic process that alters as more information becomes available. The categories were: 1) pro-death, mitochondria-dependent; 2) pro-death, mitochondria-independent; 3) autophagy; 4) pro-survival; 5) highly expressed in retina (rods, cones, Müller cells, astrocytes, bipolar cells); 6) housekeeping.

### RNA extraction and qRT-PCR analysis

All the qRT-PCR experiments complied with the MIQE (Minimum Information for Publication of Quantitative Real-Time PCR Experiments [50]) guidelines. Total RNA from retinas was extracted following standard TRIzol procedures (Invitrogen-Life Technologies, Carlsbad, CA). RNA concentration was assessed with a ND-1000 Spectrophotometer (NanoDrop Technologies, Thermo Fisher Scientific, Wilmington, DE) and RNA quality verified by microcapillary electrophoresis on an Agilent 2100 Bioanalyzer (Agilent Technologies, Santa Clara, CA) with RNA 6000 Nanochips. Only RNA with RIN> 9 and A260/280> 1.9 was used.

RNA samples were treated with RNase-free DNase and then reverse-transcribed using the High Capacity cDNA Reverse Transcription Kit following standard procedures from the manufacturer (Applied Biosystems, Foster City, CA). The qRT-PCR reactions contained 40 ng cDNA, 1x TaqMan Universal PCR master mix (Applied Biosystems), and 1x custom gene-specific TaqMan® assay or 900 nM of each unlabeled forward and reverse primer and 250 nM of FAM dye labeled TaqMan MGB probe. The SYBR green qRT-PCR reactions contained 40 ng cDNA, 1x SYBR green PCR master mix (Applied Biosystems), and 250 nM of each unlabeled forward and reverse primer. Reactions were performed in 96-well arrays using the 7500 real-time PCR machine and detection software (v2.0.1, Applied Biosystems).

Quality control and all statistical analyses were computed with RealTime StatMiner® version 4.0 (Integromics Inc., Philadelphia, PA). *GAPDH* was found to be the most stable housekeeping gene in all tested samples, and used for normalization and calculation of the ratio of diseased vs. normal using the ΔΔCT method [51]. A parametric moderated t-test, where the standard errors were moderated across all the genes using a simple Bayesian model and the p-values adjusted for the Benjamini & Hochberg (BH) step-up false discovery rate (FDR) controlling procedure, was applied to verify if the variations between groups were statistically significant. Genes with BH-adjusted p<0.05 and FC>+/- 2 were considered DE.

### Protein extraction and western blot analysis

Protein extraction and western blot were performed as previously described [27]. Briefly, for western analyses, protein extracts (30 µg) were separated by SDS-PAGE (4% stacking gel, 12% separating gel), and transferred to a polyvinylidene difluoride membrane (Trans-Blot Transfer Medium, Bio-Rad, Hercules, CA) in chilled transfer buffer. The membrane was blocked in 10% skim milk in Tris buffered saline containing 0.5% Tween-20 overnight at 4°C, and then incubated for 1.5 h with the primary antibodies. The antibodies and concentrations used are detailed in Table S4. Either ACTB or GAPDH were used as loading controls. Due to the limited availability of samples, a single retina per status/time point was analyzed. For TNFA, recombinant canine TNFA (#1507-CT, R&D Systems, Minneapolis, MN) was used as positive control and showed a band at 62 kDa, like the retinal samples, and an additional band at 17 kDa, where the monomeric form of the protein is expected. The positive controls for NFkB p65, IKKα/β, and IKBα were collected from dogs with relapsed B-cell lymphoma and used in a previously study [52].

Signal was detected by incubating with the appropriate secondary antibody conjugated with horseradish peroxidase (1:2,000, Zymed, San Francisco, CA), visualized using the ECL method according to the manufacturer's recommendations (ECL Western Blotting Detection Reagents Kit, Amersham, Piscataway, NJ), and exposed on autoradiograph films (Eastman Kodak, X-oMAT; Rochester, NY). ImageJ software (http://rsb.info.nih.gov/ij/index.html; [53]) was used to compare the intensities of the bands found with western blot analysis. Band intensities of each protein were normalized with either ACTB or GAPDH intensities.

### Fluorescent immunohistochemistry (IHC)

Seven µm cryosections of OCT embedded retinas from normal and mutant dogs were used for IHC. The procedures used for tissue collection, preparation, and sectioning were previously described [19]. Cryosections were washed and treated with the primary antibodies at dilutions listed in Table S4; both single and dual immunolabeling procedures were done. Antigen-antibody complexes were visualized with fluorochrome-labeled secondary antibodies (Alexa Fluor, 1:200, Molecular Probes, Eugene, OR) and DAPI stain was used to label cell nuclei. Slides were mounted with Gelvatol (Sigma-Aldrich, St Louis, MO) and examined either with an epifluorescent (Axioplan, Carl Zeiss Meditech, Oberkochen, Germany) or a confocal microscope (TCS SP5 spectral imaging confocal/multiphoton, Leica Microsystems, Exton, PA). Images were digitally captured (Spot 4.0 camera or Leica Application Suite LAS-AF-Lite 2.6.0, respectively), and displayed with a graphic program (Photoshop, Adobe, Mountain View, CA).

## Supporting Information

Figure S1 **Retina morphology in study models.** Morphological changes in normal, rcd1, xlpra2, and erd mutant retinas at different ages (4-4.3, 6-8, and 14.1-16 wks). PR degeneration occurs at different rates. It is more rapid and aggressive in rcd1, slightly delayed in xlpra2, while the disease in the erd model shows preservation of the ONL and no changes until at least 14.1 wks of age. Scale bar: 20 μm; RPE = retinal pigment epithelium, PR = photoreceptors, ONL = outer nuclear layer, OPL = outer plexiform layer, INL = inner nuclear layer, IPL = inner plexiform layer, GCL = ganglion cell layer.
(TIF)

Figure S2 **Protein co-localization in retina cells of study models.** Co-labeling (yellow) in normal, rcd1, and xlpra2-mutant retinas at 7 wks with antibodies against proteins expressed in different retinal cell layers (red: TNF superfamily ligands TNFA, CD40LG, and TNFSF8; TNF superfamily receptors TNFRSF1A and TNFRSF9; TNF superfamily adaptor TRADD; the initiator caspase CASP8; pro-survival molecule STAT3) and either CRALBP (green - marker for Müller cells), parvalbumin (green - marker for horizontal and amacrine cells), or Go-α (green - marker for ON bipolar cells). TNFA, CD40LG, STAT3, and TNFSF8 are expressed by Müller cells, and the latter also by horizontal and ON bipolar cells. While TNFRSF1A and CASP8 are expressed by a high number of horizontal cells, few of these cells also express TNFRSF9 and TRADD. TNFRSF9 is also expressed by amacrine cells. Single immunolabeling of these proteins at different ages is shown in Figure 6. Scale bar: 20 μm. Note that the pictures with parvalbumin were taken by confocal microscopy. A dashed line indicates that the figure was cut and an asterisk that the RPE was missing. RPE = retinal pigment epithelium, PR = photoreceptors, ONL = outer nuclear layer, OPL = outer plexiform layer, INL = inner nuclear layer, IPL = inner plexiform layer, GCL = ganglion cell layer.
(TIF)

Table S1 **List of genes tested by qRT-PCR** (modified from [18]). Genes are divided into those present in the profiling array (v4) or analyzed in single assays, and are reported with their symbols (in parenthesis the alternative symbols), descriptions, categories, TaqMan**®** assay numbers (Applied Biosystems; http://www3.appliedbiosystems.com/AB_Home/index.htm) or primer sequences. Main categories were: 1) pro-death, mitochondria-dependent; 2) pro-death, mitochondria-independent; 3) autophagy; 4) pro-survival; 5) vision related, highly expressed in retinal cells: a) PR, b) Müller cells and astrocytes, c) bipolar cells; 6) housekeeping. SYBR green was used for *FADD*, *RPGRORF15*, and *RPGRIP1*.
(DOC)

Table S2 **Western blot quantification.** ImageJ was used to quantify the signal intensities of bands found with western blot analysis for proteins that are retina-specific (RHO, SAG; Figure 3A) or involved in signaling pathways (TNFA, CD40LG, TNFSF8, TNFRSF1A, TNFRSF9, TRADD, CASP8, STAT3, NTF3, XIAP; Figure 5). Signal normalization was performed with either ACTB or GAPDH.
(XLSX)

Table S3 **Differentially expressed (DE) genes identified by qRT-PCR in study models.** DE genes (BH-adjusted p<0.05 and FC>+/-2) between rcd1, xlpra2, and erd-mutants compared to normals at different ages are divided in photoreceptor (PR) and retina-enriched or found with the signaling pathway analysis. They are listed in alphabetical order, first the up-regulated and then the down-regulated, and are reported with the FC differences compared to normals. They are separated in unique for one specific disease or common between different diseases. In red DE genes that were common for all three diseases. For the 11.9-14.1 wks erd-mutants a reduced number of genes (n=38) were tested (see Material and Methods). The complete list of genes tested is available as Table S3.
(DOC)

Table S4 **List of antibodies used for immunohistochemistry (IHC) and western blot (WB).** Antibodies are reported with the corresponding protein symbol, the source with either catalogue number/manufacturer or personal gift, descriptions, and concentrations used.
(DOC)
