# Supplementary figures and images for: Up-Regulation of Tumor Necrosis Factor Superfamily Genes in Early Phases of Photoreceptor Degeneration
Source: PLoS One. 2013 Dec 19;8(12):e85408. doi: 10.1371/journal.pone.0085408 (PMC3868615; doi:10.1371/journal.pone.0085408)

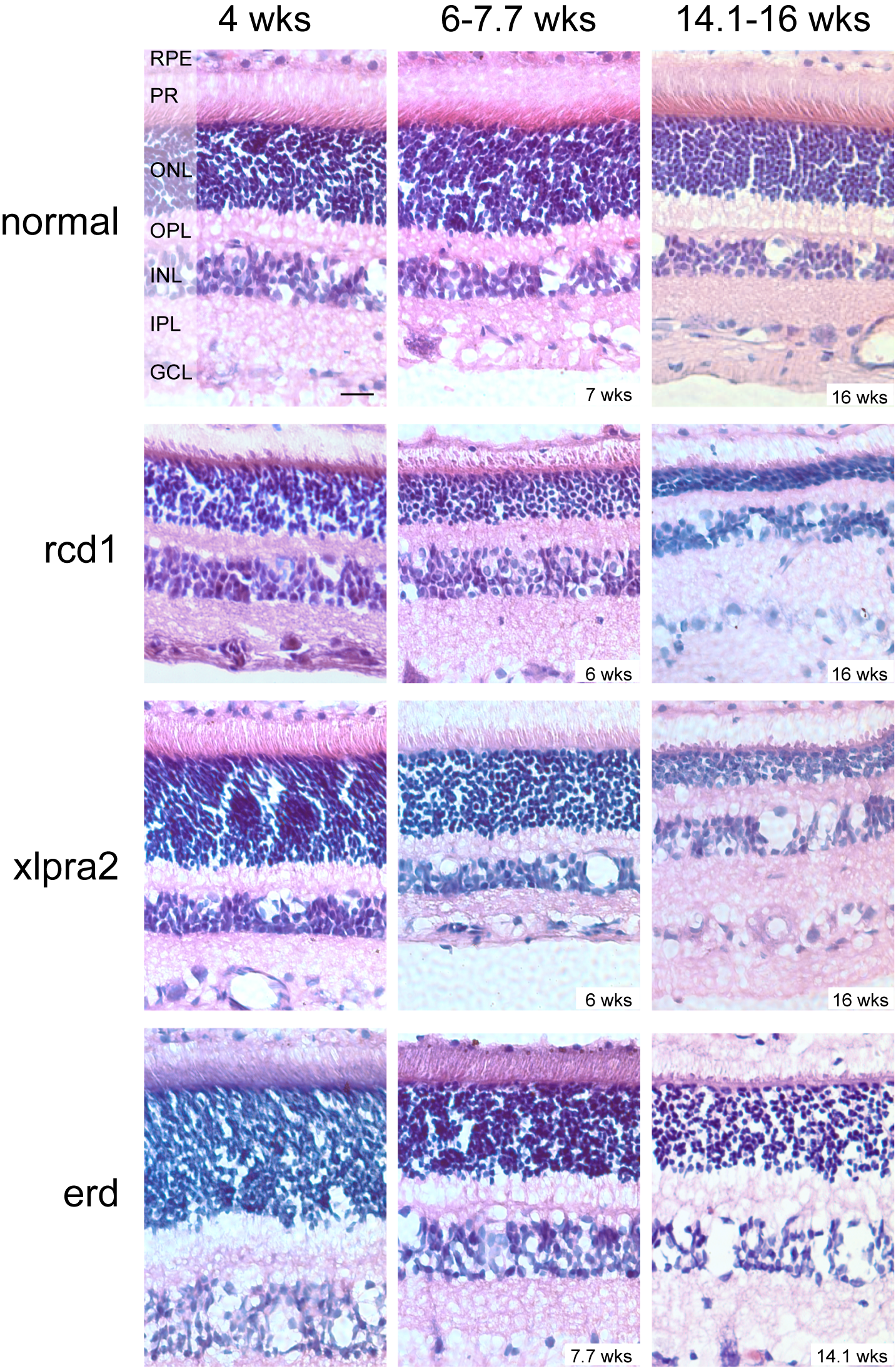

Supplement: Figure S1 — Retina morphology in study models. Morphological changes in normal, rcd1, xlpra2, and erd mutant retinas at different ages (4-4.3, 6-8, and 14.1-16 wks). PR degeneration occurs at different rates. It is more rapid and aggressive in rcd1, slightly delayed in xlpra2, while the disease in the erd model shows preservation of the ONL and no changes until at least 14.1 wks of age. Scale bar: 20 μm; RPE = retinal pigment epithelium, PR = photoreceptors, ONL = outer nuclear layer, OPL = outer plexiform layer, INL = inner nuclear layer, IPL = inner plexiform layer, GCL = ganglion cell layer. (TIF) [file pone.0085408.s001.tif]

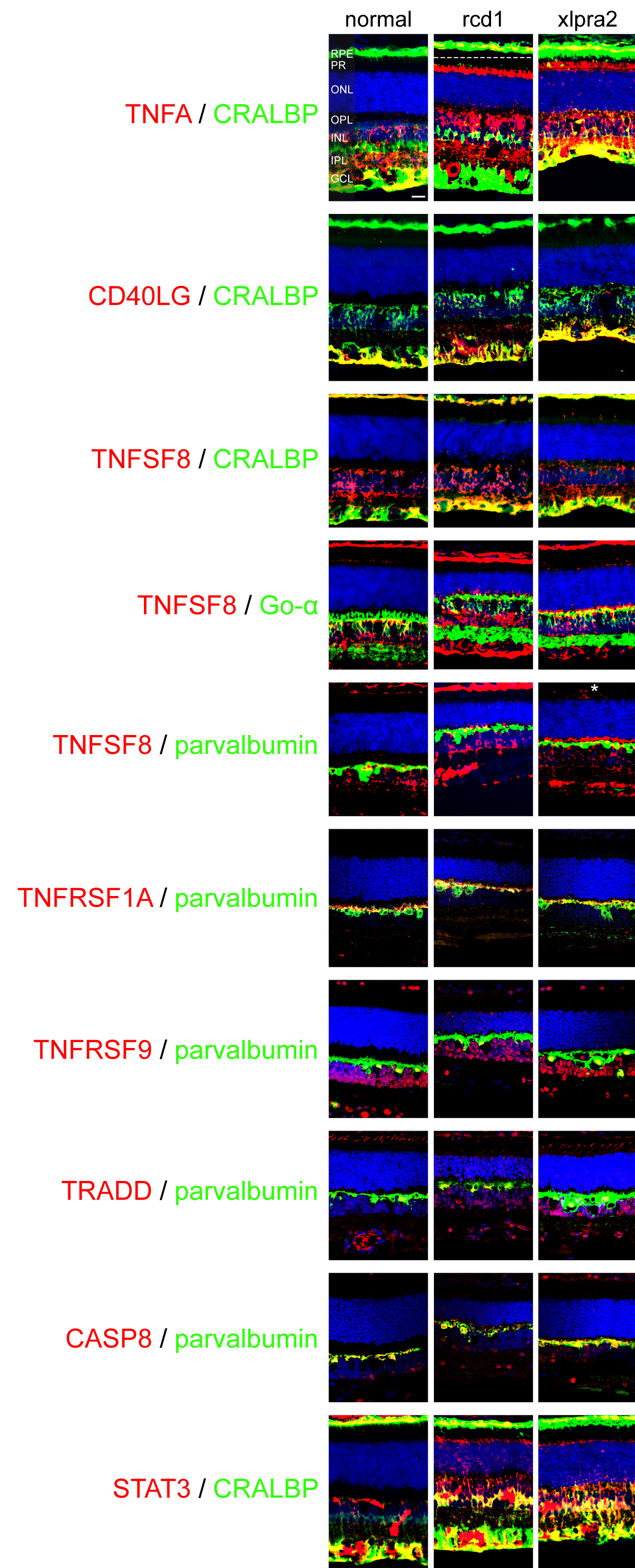

Supplement: Figure S2 — Protein co-localization in retina cells of study models. Co-labeling (yellow) in normal, rcd1, and xlpra2-mutant retinas at 7 wks with antibodies against proteins expressed in different retinal cell layers (red: TNF superfamily ligands TNFA, CD40LG, and TNFSF8; TNF superfamily receptors TNFRSF1A and TNFRSF9; TNF superfamily adaptor TRADD; the initiator caspase CASP8; pro-survival molecule STAT3) and either CRALBP (green - marker for Müller cells), parvalbumin (green - marker for horizontal and amacrine cells), or Go-α (green - marker for ON bipolar cells). TNFA, CD40LG, STAT3, and TNFSF8 are expressed by Müller cells, and the latter also by horizontal and ON bipolar cells. While TNFRSF1A and CASP8 are expressed by a high number of horizontal cells, few of these cells also express TNFRSF9 and TRADD. TNFRSF9 is also expressed by amacrine cells. Single immunolabeling of these proteins at different ages is shown in Figure 6. Scale bar: 20 μm. Note that the pictures with parvalbumin were taken by confocal microscopy. A dashed line indicates that the figure was cut and an asterisk that the RPE was missing. RPE = retinal pigment epithelium, PR = photoreceptors, ONL = outer nuclear layer, OPL = outer plexiform layer, INL = inner nuclear layer, IPL = inner plexiform layer, GCL = ganglion cell layer. (TIF) [file pone.0085408.s002.tif]
