# Supplementary material for: Up-Regulation of Tumor Necrosis Factor Superfamily Genes in Early Phases of Photoreceptor Degeneration
Source: PLoS One. 2013 Dec 19;8(12):e85408. doi: 10.1371/journal.pone.0085408 (PMC3868615; doi:10.1371/journal.pone.0085408)
Supplement: Table S1 — List of genes tested by qRT-PCR (modified from [18]). Genes are divided into those present in the profiling array (v4) or analyzed in single assays, and are reported with their symbols (in parenthesis the alternative symbols), descriptions, categories, TaqMan® assay numbers (Applied Biosystems; http://www3.appliedbiosystems.com/AB_Home/index.htm) or primer sequences. Main categories were: 1) pro-death, mitochondria-dependent; 2) pro-death, mitochondria-independent; 3) autophagy; 4) pro-survival; 5) vision related, highly expressed in retinal cells: a) PR, b) Müller cells and astrocytes, c) bipolar cells; 6) housekeeping. SYBR green was used for FADD, RPGRORF15, and RPGRIP1. (DOC) [file pone.0085408.s003.doc]

**Supplementary Table S1.** **List of genes tested by qRT-PCR** (modified from [18]). Genes are divided into those present in the profiling array (v4) or analyzed in single assays, and are reported with their symbols (in parenthesis the alternative symbols), descriptions, categories, TaqMan**®** assay numbers (Applied Biosystems; http://www3.appliedbiosystems.com/AB_Home/index.htm) or primer sequences. Main categories were: 1) pro-death, mitochondria-dependent; 2) pro-death, mitochondria-independent; 3) autophagy; 4) pro-survival; 5) vision related, highly expressed in retinal cells: a) PR, b) Müller cells and astrocytes, c) bipolar cells; 6) housekeeping. SYBR green was used for *FADD*, *RPGRORF15*, and *RPGRIP1*.

| **Gene symbol (alternative symbol)** | **Gene description** | **Category** | **TaqMan® assay or primer sequences** |
| --- | --- | --- | --- |
| ***Genes present in profiling array (v4)*** | |  |  |
| *18S* | eukaryotic 18S rRNA | 6 | Hs99999901_s1 |
| *AIFM1* (*AIF*) | apoptosis-inducing factor, mitochondrion-associated 1 | 1 | Cf02636601_m1 |
| *SLC25A4* (*ANT-1*) | solute carrier family 25, member 4 | 1 | Cf02730291_g1 |
| *APAF1* | apoptotic peptidase activating factor 1 | 1 | Cf02695305_m1 |
| *ATG3* | autophagy related 3 homolog | 3 | Cf00684119_m1 |
| *ATG5* | autophagy related 5 homolog | 3 | Cf02637561_m1 |
| *ATG7* | autophagy related 7 homolog | 3 | Cf02656560_m1 |
| *ATG12* | autophagy related 12 homolog | 3 | Cf02641158_m1 |
| *BAD* (*BBC2/BCL2L8*) | BCL2-antagonist of cell death | 1 | Cf02627333_m1 |
| *BAK1* | BCL2-antagonist/killer 1 | 1 | Cf02627218_m1 |
| *BAX* | BCL2-associated X protein | 1 | Cf02622186_g1 |
| *BBC3* (*PUMA*) | BCL2 binding component 3 | 1 | Cf02708330_m1 |
| *BCL2* | B-cell CLL/lymphoma 2 | 4 | Cf02622425_m1 |
| *BCL2L11* (*BAM/BIM*) | BCL2-like 11 | 1 | Cf00708025_s1 |
| *PABPN1* (*BCL2L2*) | poly(A) binding protein, nuclear 1 | 4 | Cf02664611_m1 |
| *BCL2L1* (*BCL-XL*) | BCL2-like 1 | 4 | Cf02622161_m1 |
| *BDNF* | brain-derived neurotrophic factor | 4 | Cf02622349_g1 |
| *BECN1* (*ATG6*) | beclin 1 | 3 | Cf02643377_m1 |
| *BID* | BH3 interacting domain death agonist | 1 | Cf03654539_m1 |
| *GRP78* (*BIP*) | 78 kDa glucose-regulated protein | 4 | Cf02631877_m1 |
| *BNIP3* (*NIP3*) | BCL2/adenovirus E1B 19kDa interacting protein 3 | 1 | Cf02654885_m1 |
| *RIPK3 (RIP3)* | receptor-interacting serine-threonine kinase 3 | 2 (necroptosis) | Cf02656149_m1 |
| *RIPK1* (*RIP1*) | receptor-interacting serine/threonine-protein kinase 1 | 2, 4 (necroptosis) | Cf02650080_m1 |
| *CASP10* | caspase 10 | 1 | Cf03460108_m1 |
| *HPRT1* | hypoxanthine phosphoribosyltransferase 1 | 6 | Cf02626256_m1 |
| *CASP2* | caspase 2 | 1 | Cf02624522_m1 |
| *CASP3* | caspase 3 | 1, 2 | Cf02622232_m1 |
| *CASP4* | caspase 4 | 1 | Cf02623472_m1 |
| *CASP6* | caspase 6 | 1 | Cf02652513_m1 |
| *CASP7* | caspase 7 | 1 | Cf03460102_m1 |
| *CASP8* | caspase 8 | 1, 2 | Cf02627553_m1 |
| *CASP9* (*APAF3*) | caspase 9 | 1, 2 | Cf02627331_m1 |
| *SFRS2IP* (*CASP11*) | splicing factor, arginine/serine-rich 2, interacting protein | 2 | Cf02703447_m1 |
| *CAPN1* | calpain 1, (mu/I) large subunit | 1, 3 | Cf02704115_m1 |
| *CAPN2* | calpain 2, (mu/II) large subunit | 1, 3 | Cf02645870_m1 |
| *CAST* | calpastatin | 2 | Cf02664849_m1 |
| *CTSD* | cathepsin D | 3 | Cf02625552_m1 |
| *CTSS* | cathepsin S | 3 | Cf02625930_m1 |
| *CCL2* | chemokine (C-C motif) ligand 2 | 4 | Cf02671955_g1 |
| *CD40* (*TNFRSF5*) | TNF receptor superfamily member 5 | 2, 4 | Cf02626290_m1 |
| *CD40LG* (*CD154*/*TNFSF5*) | CD40 ligand | 2, 4 | Cf02623314_m1 |
| *PTPRC* (*CD45*) | protein tyrosine phosphatase, receptor type C | 1 | Cf02653185_m1 |
| *CNTF* | ciliary neurotrophic factor | 4 | Cf03460095_sH |
| *CREB1* | cAMP responsive element binding protein 1 | 4 | Cf02667607_m1 |
| *CYCS* | cytochrome c, somatic | 1 | Cf02640410_g1 |
| *TYROBP* (*DAP12/KARAP*) | TYRO protein tyrosine kinase binding protein | 2 | Cf02642009_m1 |
| *DIABLO* (*SMAC*/*SMAC3*) | diablo homolog | 1 | Cf02665346_m1 |
| *ENDOG* | endonuclease G | 1 | Cf02703061_u1 |
| *INSR* (*CD220*/*HHF5*) | insulin receptor | 4 | Cf02647625_m1 |
| *FAS* (*TNFRSF6*/*APO-1*/*CD95*) | TNF receptor superfamily, member 6 | 1 | Cf02651136_m1 |
| *FASLG* (*TNFSF6*/*CD95L*/*CD178*) | FAS ligand | 1 | Cf02625215_s1 |
| *BFGF* (*FGF2*) | basic fibroblast growth factor | 4 | Cf03460065_g1 |
| *DDIT3* (*GADD153*/*CHOP10*) | DNA-damage-inducible transcript 3 | 2 | Cf02654858_m1 |
| *GAPDH* | glyceraldehyde-3-phosphate dehydrogenase | 6 | Hs02786624_g1 |
| *GDNF* | glial cell derived neurotrophic factor | 4 | Cf02691052_s1 |
| *HIF1A* | hypoxia-inducible factor 1, alpha subunit | 4 | Cf02741632_m1 |
| *HRK* (*DP5*/*HARAKIRI*) | BCL2 interacting protein | 1 | Cf02702255_g1 |
| *HSPB1* (*HSP27*) | heat shock 27kDa protein 1 | 4 | Cf02628297_m1 |
| *HSPD1* (*HSP60*) | heat shock 60kDa protein 1 (chaperonin) | 1, 4 | Cf02668830_gH |
| *HSP70* (*HSPA1*) | heat shock protein 70 | 4 | Cf02622418_g1 |
| *HSP86* (*HSP90AA1*) | heat shock protein HSP90-alpha | 4 | Cf03460183_s1 |
| *IGF1R* (*CD221*) | insulin-like growth factor 1 receptor | 4 | Cf02625178_m1 |
| *IL6* (*IFNB2*) | interleukin 6 (interferon, beta 2) | 2, 4 | Cf02624282_m1 |
| *IL10* | interleukin 10 | 4 | Cf02624265_m1 |
| *MAP1LC3A* (*LC3*) | microtubule-associated protein 1 light chain 3 alpha | 3 | Cf02630406_m1 |
| *LYZ* | lysozyme | 3 | Cf02642933_m1 |
| *PRKCZ* (*PKC2*) | protein kinase C, zeta | 4 | Cf02674616_m1 |
| *PRDX3* | Peroxiredoxin 3 | 4 | Cf03460191_sH |
| *NGF (NGFB)* | nerve growth factor (beta polypeptide) | 4 | Cf02625041_s1 |
| *NTF3* | neurotrophin 3 | 4 | Cf02700489_s1 |
| *NTF4* | neurotrophin 4 | 4 | Cf02705704_s1 |
| *SOD1* | superoxide dismutase 1, soluble | 1, 4 | Cf02624276_m1 |
| *STAT1* | signal transducer and activator of transcription 1 | 1 | Cf02662970_m1 |
| *STAT3* | signal transducer and activator of transcription 3 | 4 | Cf02666647_m1 |
| *BIRC5* (*IAP4*) | baculoviral IAP repeat-containing 5 (survivin) | 4 | Cf02628995_m1 |
| *TNFA* | tumor necrosis factor alpha | 1, 2, 4 | Cf02628236_m1 |
| *TNFRSF1A* | tumor necrosis factor receptor superfamily, member 1A | 1, 2, 4 | Cf02622751_m1 |
| *CD18* (*ITGB2*) | integrin, beta 2 | 2, 4 | Cf02623835_m1 |
| *TNFRSF25* (*APO-3*/*DDR3*) | tumor necrosis factor receptor superfamily, member 25 | 1, 2 | Cf02653814_g1 |
| *TNFSF10* (*APO-2L*/*TRAIL*) | tumor necrosis factor (ligand) superfamily, member 10 | 1, 2 | Cf03460069_m1 |
| *TNFRSF9* (*4-1BB*/*CD137*) | tumor necrosis factor receptor superfamily, member 9 | 2, 4 | Cf03460132_m1 |
| *TNFSF8* (*CD153*/*CD30L*) | tumor necrosis factor (ligand) superfamily, member 8 | 2, 4 | Cf03460158_m1 |
| *TP53* | tumor protein p53 | 1 | Cf02623148_m1 |
| *TP73* | tumor protein p73 | 1, 4 | Cf02680478_mH |
| *TRADD* | TNFRSF1A-associated via death domain | 1, 2, 4 | Cf02661903_m1 |
| *TRAF2* (*TRAP*) | TNF receptor-associated factor 2 | 4 | Cf02662893_m1 |
| *TRAF3* | TNF receptor-associated factor 3 | 2 | Cf02659700_m1 |
| *XIAP* (*AP13*/*BIRC4*) | X-linked inhibitor of apoptosis | 4 | Cf02625207_m1 |
| *ACTB* | actin, beta | 6 | Hs03023880_g1 |
| *RHO* | rhodopsin | 5a | Cf02625669_m1 |
| *OPN1SW/S-opsin* | opsin 1 (cone pigments), short-wave-sensitive, blue opsin | 5a | Cf03460200_m1 |
| *OPN1LW/L-opsin* | opsin 1 (cone pigments), long-wave-sensitive, red/green opsin | 5a | Cf02622926_m1 |
| *ARR3* (*CAR*/*ARRX*) | retinal cone arrestin 3 (X-arrestin) | 5a | Cf03460116_m1 |
| *VIM* | vimentin | 5b | Cf02668853_g1 |
| *GFAP* | glial fibrillary acidic protein | 5b | Cf02655695_m1 |
| *PRKCA* (*PKCA*) | protein kinase C, alpha | 2, 5c | Cf02655322_m1 |
|  |  |  |  |
|  |  |  |  |
| ***Genes analyzed with single assays*** | |  |  |
| *PLAGL2 (ZNF900)* | pleiomorphic adenoma-like protein 2 | 1 | **F:** GGCTGCCTCTCTCCACTGAA  **R:** AGGCAAATGAGAAGTGGTGTTTC  **TaqMan:** ATTCGGCACCAGAAGT |
| *EDN2* (*ET2*) | endothelin 2 | 4 | Cf02622240_m1 |
| *EDNRB* (*ETB*) | endothelin receptor type B | 4 | **F**: AAGACAGCTAAAGATTGGTGGCTATT  **R**: CAACATTTCACAGGTCATCAGGGTATA  **TaqMan**: ATGGCAAGCAGAAATA |
| *FADD* (*GIG3*/*MORT1*) | FAS-associating death domain-containing protein | 1 | **F**: CTGGTGGCCGACCTCATC  **R**: CCTCTACTGCTCTTGCTCTCATTCT |
| *ZBTB4(ZNF903)* | zinc finger and BTB domain containing 4 | 2 | Cf02713017_g1 |
| *RPGRIP1* (*LCA6/CORD13/RGI1*) | X-linked retinitis pigmentosa GTPase regulator-interacting protein 1 | 5a | **F**: ACCACACTTACTGGAGCTGGTGGAGAA  **R**: GGAGGCCGTGGCATCTGATGA |
| *NFKB1* (*p50*/*p105*/*KBF1*/*EBP-1*) | nuclear factor of kappa light polypeptide gene enhancer in B-cells 1 | 1, 4 | Cf02622551_m1 |
| *BNIP3L* (*NIX*) | BCL2/adenovirus E1B 19kDa-interacting protein 3-like | 1 | Cf03460134_m1 |
| *CRX (CORD2/CRD/LCA7, OTX3)* | cone-rod homeobox | 5a | Cf02624491_mH |
| *SAG (RP47/S-AG)* | S-antigen; retina and pineal gland (arrestin) | 5a | Cf02628845_m1 |
| *CNGB3* | cyclic nucleotide gated channel beta 3 | 5a | **F**: AAGATCCTGATCCAAGCAATCAG  **R**: CTTCAAACGTGACTGGAGTCATCT  **TaqMan**: CTCAGCAATCTACAAGACA |
| *CNGA3 (ACHM2/CCNC1/CNCG3/CNG3)* | cyclic nucleotide gated channel alpha 3 | 5a | **F**: GCCCTGCCTGTCTTCTATAACTG  **R**: CACAGCATCACGTGCTCAGA  **TaqMan**: TGTGCAGGGCCTGTT |
| *RPGR (COD1/CORDX1/CRD/PCDX/RP15/RP3/XLRP3)* | X-linked retinitis pigmentosa GTPase regulator; retinitis pigmentosa GTP-ase regulator | 5a | **F**: GGAACCCGAGGAGGTGATG  **R**: CAGCAAATTGAGTTTTCCCAAAT  **TaqMan**: AGTTCAGGTGCTGTGTTT |
| *NDUFS4 (AQDQ/CI-18)* | NADH dehydrogenase (ubiquinone) Fe-S protein 4, 18kDa (NADH-coenzyme Q reductase) | 1 | Cf02644388_g1 |
| *SLC25A5 (2F1/AAC2/ANT2/T2/T3)* | solute carrier family 25 (mitochondrial carrier; adenine nucleotide translocator), member 5 | 4 | Cf02740079_gH |
| *CNGB1 (CNCG2/CNCG3L/CNCG4/CNGB1B/GAR1/GARP/RCNC2/RCNCbeta/RP45)* | cyclic nucleotide gated channel beta 1 | 5a | **F**: TGCATTATCCTGAGTCTCAGAAGTTAC  **R**: TCCTTGGGCTTGTTGTTATTTCTC  **TaqMan**: AGAAGGCCAGGCGCA |
| *CNGA1 (CNCG/CNCG1/CNG1/RCNC1/RCNCalpha/RP49)* | cyclic nucleotide gated channel alpha 1 | 5a | **F**: ATGAAACCCCCCATGCAA  **R**: TGGCACCAGGCAGGTACTG  **TaqMan**: ATCCATCACAGAGGGA |
| *RPGRORF15* | X-linked retinitis pigmentosa GTPase regulator; retinitis pigmentosa GTP-ase regulator ORF15 | 5a | **F**: AGGGGAAAGAGCGTGAGGCACA  **R**: ACATTAGTCCAGAATTTTTTAGAACCCGG |
