# Supplementary material for: Up-Regulation of Tumor Necrosis Factor Superfamily Genes in Early Phases of Photoreceptor Degeneration
Source: PLoS One. 2013 Dec 19;8(12):e85408. doi: 10.1371/journal.pone.0085408 (PMC3868615; doi:10.1371/journal.pone.0085408)
Supplement: Table S3 — Differentially expressed (DE) genes identified by qRT-PCR in study models. DE genes (BH-adjusted p<0.05 and FC>+/-2) between rcd1, xlpra2, and erd-mutants compared to normals at different ages are divided in photoreceptor (PR) and retina-enriched or found with the signaling pathway analysis. They are listed in alphabetical order, first the up-regulated and then the down-regulated, and are reported with the FC differences compared to normals. They are separated in unique for one specific disease or common between different diseases. In red DE genes that were common for all three diseases. For the 11.9-14.1 wks erd-mutants a reduced number of genes (n=38) were tested (see Material and Methods). The complete list of genes tested is available as Table S3. (DOC) [file pone.0085408.s005.doc]

**Supplementary Table S3. Differentially expressed (DE) genes identified by qRT-PCR in study models.** DE genes (BH-adjusted p<0.05 and FC>+/-2) between rcd1, xlpra2, and erd-mutants compared to normals at different ages are divided in photoreceptor (PR) and retina-enriched or found with the signaling pathway analysis. They are listed in alphabetical order, first the up-regulated and then the down-regulated, and are reported with the FC differences compared to normals. They are separated in unique for one specific disease or common between different diseases. In red DE genes that were common for all three diseases. For the 11.9-14.1 wks erd-mutants a reduced number of genes (n=38) were tested (see Material and Methods). The complete list of genes tested is available as Supplementary Table S1.

| **DE genes unique rcd1 (n=8)** | **FC** |  |  |  |  |  |  |  |  |
| --- | --- | --- | --- | --- | --- | --- | --- | --- | --- |
| ***3 wks*** |  |  |  |  |  |  |  |  |  |
| ***Signaling pathway analysis*** | | |  |  |  |  |  |  |  |
| *BBC3* | 2.3 |  |  |  |  |  |  |  |  |
| *CAPN1* | 2.0 |  |  |  |  |  |  |  |  |
| *CASP8* | 2.3 |  |  |  |  |  |  |  |  |
| *CCL2* | 3.9 |  |  |  |  |  |  |  |  |
| *CD40LG* | 2.6 |  |  |  |  |  |  |  |  |
| *FAS* | 2.2 |  |  |  |  |  |  |  |  |
| *IL6* | 2.3 |  |  |  |  |  |  |  |  |
| *TNFA* | 3.5 |  |  |  |  |  |  |  |  |
| **DE genes unique rcd1 (n=18)** | **FC** |  |  |  |  |  |  |  |  |
| ***5 wks*** |  |  |  |  |  |  |  |  |  |
| ***PR and retina-enriched*** | | |  |  |  |  |  |  |  |
| *GFAP* | 9.6 |  |  |  |  |  |  |  |  |
| *ARR3* | -2.4 |  |  |  |  |  |  |  |  |
| *CNGA1* | -3.3 |  |  |  |  |  |  |  |  |
| *RHO* | -2.8 |  |  |  |  |  |  |  |  |
| *RPGRIP1* | -2.1 |  |  |  |  |  |  |  |  |
| *SAG* | -2.0 |  |  |  |  |  |  |  |  |
| ***Signaling pathway analysis*** | | |  |  |  |  |  |  |  |
| *CASP4* | 2.4 |  |  |  |  |  |  |  |  |
| *CASP8* | 2.1 |  |  |  |  |  |  |  |  |
| *CCL2* | 6.9 |  |  |  |  |  |  |  |  |
| *EDN2* | 2.2 |  |  |  |  |  |  |  |  |
| *FAS* | 2.6 |  |  |  |  |  |  |  |  |
| *NTF3* | 6.1 |  |  |  |  |  |  |  |  |
| *PTPRC* | 2.0 |  |  |  |  |  |  |  |  |
| *STAT3* | 3.4 |  |  |  |  |  |  |  |  |
| *TNFA* | 2.6 |  |  |  |  |  |  |  |  |
| *TNFRSF1A* | 7.7 |  |  |  |  |  |  |  |  |
| *PRDX3* | -2.9 |  |  |  |  |  |  |  |  |
| *XIAP* | -2.4 |  |  |  |  |  |  |  |  |
| **DE genes unique rcd1 (n=28)** | **FC** | **DE genes unique xlpra2 (n=1)** | **FC** | **DE genes common rcd1/xlpra2 (n=20)** | **FC** | **DE genes common 7 wks rcd1/8.3-9.9 wks erd (n=7)** | **FC** | **DE genes common 7 wks xlpra2/8.3-9.9 wks erd (n=7)** | **FC** |
| ***7 wks*** |  |  |  |  |  |  |  |  |  |
| ***PR and retina-enriched*** | | |  |  |  |  |  |  |  |
| *ARR3* | -2.2 |  |  | *CNGB3* | 2.5/2.3 | *GFAP* | 14.6/3.4 | *GFAP* | 4.6/3.4 |
| *CNGA1* | -3.0 |  |  | *GFAP* | 14.6/4.6 |  |  |  |  |
| *RPGRIP1* | -3.4 |  |  | *RHO* | -3.6/-2.3 |  |  |  |  |
| *SAG* | -2.9 |  |  |  |  |  |  |  |  |
| ***Signaling pathway analysis*** | | |  |  |  |  |  |  |  |
| *BAK1* | 2.6 | *CASP3* | 2.1 | *ATG5* | 2.0/2.0 | *CD40LG* | 30.9/3.8 | *CD40LG* | 15.1/3.8 |
| *BBC3* | 3.1 |  |  | *CASP8* | 2.8/2.1 | *HSP90* | 2.0/2.2 | *HSP90* | 2.1/2.2 |
| *BCL2* | 2.9 |  |  | *CCL2* | 16.1/6.1 | *STAT3* | 5.1/3.7 | *STAT3* | 2.4/3.7 |
| *BDNF* | 3.1 |  |  | *CD40LG* | 30.9/15.1 | *TNFA* | 11.2/5.3 | *TNFA* | 4.3/5.3 |
| *CAPN2* | 2.3 |  |  | *HSP90* | 2.0/2.1 | *TNFRSF1A* | 26.3/9.3 | *TNFRSF1A* | 9.1/9.3 |
| *CASP4* | 4.5 |  |  | *IL10* | 3.8/2.0 | *TNFRSF9* | 5.1/3.3 | *TNFRSF9* | 4.0/3.3 |
| *CNTF* | 3.4 |  |  | *ITGB2* | 3.0/2.5 |  |  |  |  |
| *CTSS* | 2.7 |  |  | *LYZ* | 3.1/2.2 |  |  |  |  |
| *EDN2* | 3.4 |  |  | *NTF3* | 32.1/2.7 |  |  |  |  |
| *FADD* | 2.7 |  |  | *PTPRC* | 5.8/3.4 |  |  |  |  |
| *FAS* | 2.9 |  |  | *STAT3* | 5.1/2.4 |  |  |  |  |
| *FASLG* | 4.5 |  |  | *TNFA* | 11.2/4.3 |  |  |  |  |
| *GDNF* | 3.0 |  |  | *TNFRSF1A* | 26.3/9.1 |  |  |  |  |
| *HRK* | 10.9 |  |  | *TNFSF8* | 14.5/9.2 |  |  |  |  |
| *HSPB1* | 2.0 |  |  | *TNFRSF9* | 5.1/4.0 |  |  |  |  |
| *IL6* | 5.3 |  |  | *TRADD* | 2.6/2.9 |  |  |  |  |
| *MAP1LC3A* | 2.2 |  |  | *TYROBP* | 3.1/2.5 |  |  |  |  |
| *NGF* | 3.5 |  |  |  |  |  |  |  |  |
| *NTF4* | 4.9 |  |  |  |  |  |  |  |  |
| *SOD1* | 2.0 |  |  |  |  |  |  |  |  |
| *TNFRSF25* | 2.8 |  |  |  |  |  |  |  |  |
| *TRAF3* | 2.0 |  |  |  |  |  |  |  |  |
| *SLC25A5* | -2.2 |  |  |  |  |  |  |  |  |
| *XIAP* | -2.0 |  |  |  |  |  |  |  |  |
| **DE genes unique rcd1 (n=13)** | **FC** | **DE genes unique xlpra2 (n=6)** | **FC** | **DE genes common rcd1/xlpra2 (n=35)** | **FC** | **DE genes common 16 wks rcd1/11.9-14.1 wks erd (n=14)** | **FC** | **DE genes common 16 wks xlpra2/11.9-14.1 wks erd (n=11)** | **FC** |
| ***16 wks*** |  |  |  |  |  |  |  |  |  |
| ***PR and retina-enriched*** | | |  |  |  |  |  |  |  |
| *VIM* | 2.0 |  |  | *GFAP* | 19.7/8.4 | *GFAP* | 19.7/13.6 | *GFAP* | 8.4/13.6 |
| *PRKCA* | -2.3 |  |  | *OPN1SW* | -4.9/-2.5 | *CNGA1* | -2.0/-2.1 | *RHO* | -4.5/-2.2 |
|  |  |  |  | *RHO* | -9.8/-4.5 | *CRX* | -2.0/-2.0 | *SAG* | -3.9/-2.4 |
|  |  |  |  | *SAG* | -6.4/-3.9 | *RPGRIP1* | -2.0/-3.1 |  |  |
|  |  |  |  |  |  | *RHO* | -9.8/-2.2 |  |  |
|  |  |  |  |  |  | *SAG* | -6.4/-2.4 |  |  |
| ***Signaling pathway analysis*** | | |  |  |  |  |  |  |  |
| *BBC3* | 2.8 | *EDN2* | 2.7 | *CASP3* | 2.1/2.0 | *CCL2* | 43.2/12.7 | *CCL2* | 15.1/12.7 |
| *BDNF* | 2.0 | *HSP90* | 2.2 | *CASP4* | 12.0/5.6 | *CD40LG* | 8.2/4.0 | *CD40LG* | 11.8/4.0 |
| *CAPN1* | 2.6 | *HSPB1* | 2.2 | *CASP8* | 8.2/4.3 | *IL6* | 26.8/3.3 | *IL6* | 7.3/3.3 |
| *CAPN2* | 2.4 | *LYZ* | 2.4 | *CCL2* | 43.2/15.1 | *PTPRC* | 16.4/3.4 | *PTPRC* | 8.9/3.4 |
| *CD40* | 2.3 | *TRAF3* | 2.2 | *CD40LG* | 8.2/11.8 | *STAT3* | 4.7/3.3 | *STAT3* | 4.3/3.3 |
| *CNTF* | 3.2 | *SLC25A5* | -2.3 | *CTSS* | 5.2/3.1 | *TNFA* | 25.0/5.9 | *TNFA* | 9.5/5.9 |
| *MAP1LC3A* | 2.2 |  |  | *FADD* | 3.6/2.4 | *TNFRSF1A* | 7.3/4.1 | *TNFRSF1A* | 8.8/4.1 |
| *NDUFS4* | 2.0 |  |  | *FAS* | 8.8/3.2 | *TNFRSF9* | 3.2/4.1 | *TNFRSF9* | 3.9/4.1 |
| *NTF4* | 4.9 |  |  | *FASLG* | 7.5/5.0 |  |  |  |  |
| *PRDX3* | 2.5 |  |  | *GDNF* | 3.3/2.1 |  |  |  |  |
| *TNFRSF25* | 2.9 |  |  | *HRK* | 8.6/8.1 |  |  |  |  |
|  |  |  |  | *IGF1R* | 2.4/2.4 |  |  |  |  |
|  |  |  |  | *IL10* | 9.6/6.1 |  |  |  |  |
|  |  |  |  | *IL6* | 26.8/7.3 |  |  |  |  |
|  |  |  |  | *ITGB2* | 4.2/4.3 |  |  |  |  |
|  |  |  |  | *NFKB1* | 3.4/2.0 |  |  |  |  |
|  |  |  |  | *NGF* | 3.5/2.6 |  |  |  |  |
|  |  |  |  | *NTF3* | 42.2/48.8 |  |  |  |  |
|  |  |  |  | *PTPRC* | 16.4/8.9 |  |  |  |  |
|  |  |  |  | *RIPK1* | 3.4/2.6 |  |  |  |  |
|  |  |  |  | *RIPK3* | 9.5/3.2 |  |  |  |  |
|  |  |  |  | *STAT1* | 7.6/4.1 |  |  |  |  |
|  |  |  |  | *STAT3* | 4.7/4.3 |  |  |  |  |
|  |  |  |  | *TNFA* | 25.0/9.5 |  |  |  |  |
|  |  |  |  | *TNFRSF1A* | 7.3/8.8 |  |  |  |  |
|  |  |  |  | *TNFRSF9* | 3.2/3.9 |  |  |  |  |
|  |  |  |  | *TNFSF10* | 9.3/4.5 |  |  |  |  |
|  |  |  |  | *TNFSF8* | 16.2/9.0 |  |  |  |  |
|  |  |  |  | *TRADD* | 3.2/3.7 |  |  |  |  |
|  |  |  |  | *TYROBP* | 13.6/6.5 |  |  |  |  |
|  |  |  |  | *XIAP* | -2.3/-2.0 |  |  |  |  |
