# Supplementary material for: Up-Regulation of Tumor Necrosis Factor Superfamily Genes in Early Phases of Photoreceptor Degeneration
Source: PLoS One. 2013 Dec 19;8(12):e85408. doi: 10.1371/journal.pone.0085408 (PMC3868615; doi:10.1371/journal.pone.0085408)
Supplement: Table S4 — List of antibodies used for immunohistochemistry (IHC) and western blot (WB). Antibodies are reported with the corresponding protein symbol, the source with either catalogue number/manufacturer or personal gift, descriptions, and concentrations used. (DOC) [file pone.0085408.s006.doc]

**Supplementary Table S4.** **List of antibodies used for immunohistochemistry (IHC) and western blot (WB).** Antibodies are reported with the corresponding protein symbol, the source with either catalogue number/manufacturer or personal gift, descriptions, and concentrations.

| **Protein*** | **Source or company**  **(catalog #)**** | **Description** | | **IHC (concentration)** | **WB (concentration)** |
| --- | --- | --- | --- | --- | --- |
| RHO | Millipore (MAB5316) | | Mouse monoclonal | 1/1,000 | - |
|  | Millipore (MABN15) | | Mouse monoclonal | - | 1/1,000 |
| TNFA | R&D Systems (MAB1507) | | Mouse monoclonal | 1/200 | 1/100 |
| XIAP | Cell Signaling (2045) | | Rabbit monoclonal | 1/100 | 1/250 |
| TNFRSF1A | Abcam (ab19139) | | Rabbit polyclonal | 1/3,000 | 1/5,000 |
| NT3 | Santa Cruz Biotechnology (sc-547) | | Rabbit polyclonal | 1/250 | 1/100 |
| STAT3 | Cell Signaling (9139) | | Mouse monoclonal | 1/250 | 1/500 |
| ARR3 (hCAR) | Provided by C. Craft | | Rabbit polyclonal | 1/10,000 | - |
| SAG (S-antigen) | Millipore (MAB5580) | | Mouse monoclonal | 1/500 | 1/500 |
| TNFRSF9 | Aviva (ARP60755_P050) | | Rabbit polyclonal | 1/500 | 1/1,500 |
| CD40LG | Aviva (ARP33831_P050) | | Rabbit polyclonal | 1/250 | 1/750 |
| TRADD | Aviva (AVARP02012_P050) | | Rabbit polyclonal | 1/250 | 1/500 |
| TNFSF8 | Aviva (ARP63790_P050) | | Rabbit polyclonal | 1/500 | 1/1,500 |
| Active-CASP8 | Assay Biotechnology (B0059) | | Rabbit polyclonal | 1/100 | 1/1,000 |
| Phospho-NFkB p65 | Cell Signaling (3033) | | Rabbit monoclonal | 1/50 | 1/200 |
| Phospho-IKKα/β | Cell Signaling (2697) | | Rabbit monoclonal | 1/50 | 1/200 |
| Phospho-IkBα | Cell Signaling (2859) | | Rabbit monoclonal | 1/50 | 1/200 |
| Parvalbumin | Millipore (MAB1572) | | Mouse monoclonal | 1/2,000 | - |
| Go-alpha | Millipore (MAB3073) | | Mouse monoclonal | 1/5,000 | - |
| CRALBP (co-labeling with TNFA or STAT3) | Provided by J. Saari | | Rabbit polyclonal | 1/500 | - |
| CRALBP (co-labeling with CD40LG, TNFRSF9, or TNFSF8) | Thermo Fisher Scientific (MA1-813) | | Mouse monoclonal | 1/50 | - |
| ACTB | Millipore (MAB1501) | | Mouse monoclonal | - | 1/10,000 |
| GAPDH | Millipore (MAB374) | | Mouse monoclonal | - | 1/50,000 |

*: Antibodies for NT4 (sc-545, Santa Cruz Biotechnology), FAS (05-201, Upstate Cell Signaling Solutions, Temecula, CA), CASP8 (A0059 and L0167, Assay Biotechnology), CASP3 (9661, Cell Signaling; AF835, R&D Systems; ab4051, Abcam; L0103 and L0104, Assay Biotechnology), and TNFA (55383, AnaSpec, Fremont, CA; AF1507, R&D Systems) showed no specificity and did not give consistent results in dog either by IHC or WB following the protocols described in Materials and Methods.

**: the commercial sources for antibodies were: Assay Biotechnology Company Inc., Sunnyvale, CA; Aviva Systems Biology Corporation, San Diego, CA; Abcam, Cambridge, MA; Cell Signaling, Danvers, MA; Millipore, Billerica, MA; R&D Systems, Minneapolis, MN; Santa Cruz Biotechnology, Santa Cruz, CA; Thermo Fisher Scientific Inc., Pierce Antibodies, Rockford, IL.
